# HIV Tat-activated microglial extracellular vesicles induce neuronal iron dysregulation and synaptodendritic injury

**DOI:** 10.1186/s12964-026-03032-6

**Published:** 2026-06-29

**Authors:** Uma Maheswari Deshetty, Frida L. Martínez-Cuevas, Poojashree Chettiar, Shashank M Dravid, Palsamy Periyasamy, Susmita Sil, Shilpa Buch

**Affiliations:** 1 Department of Pharmacology and Experimental Neuroscience, University of Nebraska Medical Center, Omaha, Nebraska USA; 2 Department of Psychiatry and Behavioral Sciences, Texas A&M University, College Station, Texas USA

**Keywords:** Extracellular vesicles, Iron dysregulation, Synaptodegeneration, Mitochondrial dysfunction, HIV-1 Tat

## Abstract

Extracellular vesicles (EVs) are membrane-enclosed, nanoscale structures released by cells and play a key role in intercellular communication under both normal physiological and pathological conditions. They serve as conduits for transferring molecular cargo between neighboring cells, thereby modulating recipient cell function. While the HIV Transactivator of transcription (Tat) protein has been shown to induce ferroptosis in microglia, the role of Tat-activated microglia-derived EVs (Tat-MEVs) in transferring iron-handling and ferroptosis-associated cargo to neurons and promoting neuronal injury remains unexplored. In this study, we sought to evaluate the impact of cargo derived from Tat-MEVs on neuronal synaptodendritic degeneration. Rat primary cortical and hippocampal neurons were exposed to either control MEVs or Tat-MEVs and subsequently assessed for synaptodendritic degeneration, expression of key ferroptotic mediators, and mitochondrial dysfunction associated with neuronal injury. Neurons exposed to Tat-MEVs demonstrated increased expression of the key iron-handling and ferroptosis-associated proteins (transferrin, TF; transferrin receptor 1, TFR1; Six-Transmembrane Epithelial Antigen of the Prostate 3, STEAP3; divalent metal transporter 1, DMT1; and ferritin heavy chain 1, FTH1); inhibitory synaptic markers (GAD65, Gephyrin), Fe^2+^/total iron content, neuronal cytotoxicity and mitochondrial reactive oxygen species (ROS) compared to neurons exposed to control MEVs. These findings suggest a link between mitochondrial dysfunction and neuronal iron accumulation. The expression of these mediators was downregulated in neurons exposed to MEVs derived from iron chelator, deferoxamine (DFO)-pretreated BV2 cells.

Electrophysiological recordings further revealed reduced miniature excitatory postsynaptic currents in neurons exposed to Tat-MEVs, an effect that was attenuated in neurons exposed to DFO-derived MEVs. Additionally, dendritic spine analyses of neurons exposed to Tat MEVs revealed a reduction in mushroom and stubby spine subtypes, suggesting synaptodendritic injury.

Collectively, these findings demonstrate that Tat-MEVs transfer iron-handling and ferroptosis-associated cargo that promotes neuronal iron dysregulation, oxidative stress, mitochondrial dysfunction, and synaptodendritic degeneration. These changes are consistent with ferroptosis-associated neuronal stress and contribute to functional impairment in recipient neurons. This EV-based communication axis provides mechanistic insight into how HIV Tat-induced microglial dysfunction propagates iron-dependent neurotoxic signaling within the central nervous system and identifies EV-mediated iron dysregulation as a potential therapeutic target in NeuroHIV.

**Supplementary Information:**

The online version contains supplementary material available at https://doi.org/10.1186/s12964-026-03032-6.

## Introduction

Human immunodeficiency virus-1 (HIV) infection has emerged as a global health concern over the past two decades, with approximately 40 million individuals living with HIV-1 worldwide as of 2023. Although the advent of combinatorial antiretroviral therapy (cART) has significantly reduced mortality and extended the lifespan of people living with HIV (PLWH), neurological complications, collectively referred to as NeuroHIV, remain prevalent in PLWH [1–3]. Despite the ability of cART regimen to dramatically reduce plasma viremia, the incidence and prevalence of cognitive impairments remain alarmingly high in PLWH [4]. It has been suggested that almost 10–20% of PLWH are afflicted with varying degrees of neurodegeneration, thereby suggestive that despite cART, likely persistence of ongoing low-level virus replication and that of the early viral protein, such as HIV Tat in the central nervous system (CNS), could likely be contributing to the development and progression of NeuroHIV [5, 6]. Persistent viral proteins such as Tat, glycoprotein (gp120), and Nef are known to induce neurotoxicity by downregulating synaptodendritic arborization and disrupting intracellular iron homeostasis [7–9].

Elevated serum and intracellular iron levels are reported to worsen HIV pathogenesis, and HIV-driven immune dysregulation, including CD4 + T-cell impairment, has been associated with altered expression of iron-related proteins in NeuroHIV [10–13]. Viruses can hijack host iron metabolism to facilitate their replication, disrupting iron homeostasis and promoting dysfunction of organelles including mitochondria and lysosomes[ 12, 14–16]. Furthermore, viral proteins such as Tat have been shown to disrupt the structure and function of mitochondria and endolysosome compartments [17–21], causing endolysosomal deacidification and membrane destabilization, thereby facilitating iron release from endolysosomal stores into the extracellular space [17, 20–23].

Consequently, this disrupts mitochondrial function, leading to increased mitochondrial iron accumulation, elevated reactive oxygen species (ROS) production, and ultimately neuronal injury and cell death. Emerging evidence suggests that ferroptosis-associated mechanisms may contribute to the pathogenesis of NeuroHIV [24, 25].

Ferroptosis is an emerging form of regulated cell death controlled by multiple pathways, including the iron metabolic pathway, lipid peroxidation pathway, glutathione peroxidase 4 (GPX4) synthesis pathway, and the FSP1-CoQ10-NAD(P)H system [26]. Extracellular ferric iron (Fe^3+^) binds to the transferrin (TF, iron transport protein) and is internalized via transferrin receptor 1 (TFR1)-mediated endocytosis into the endosomes. Within endosomes, Fe^3+^ iron is reduced to ferrous iron (Fe^2+^) by six-transmembrane epithelial antigen of prostate (STEAP3) protein and subsequently transported into the cytosolic labile iron pool by divalent metal transporter 1 (DMT1) [27]. Endosomes, therefore, play a critical role in maintaining intracellular iron (Fe²⁺) homeostasis. Dysregulation of iron within endosomes, the cytosol, or mitochondria promotes Fenton chemistry, leading to the generation of ROS, which are implicated in the pathogenesis of NeuroHIV [22, 28, 29]. Previous studies from our group [30] and others [31, 32] have demonstrated that HIV Tat induces ROS production, resulting in oxidative stress and activation of ferroptotic pathways in microglia.

Under normal physiological conditions, microglia and astrocytes are known to release extracellular vehicles (EVs) that carry cargoes supporting neuronal metabolic functions essential for synaptic plasticity [33–37]. Notably, neurons are not directly infected by HIV-1; instead, microglia and astrocytes, to some extent, are the key cells supporting virus replication in the CNS. There is ample evidence by us [38–40] and others [41, 42] that glial cells exposed to HIV or HIV proteins release EVs carrying inflammatory cargoes, which, in turn, can be taken up by the neurons to mediate neuronal damage. Our previous study demonstrated that HIV Tat-activated astrocyte-derived EVs containing miR-7 were taken up by rat primary hippocampal neurons, leading to neuronal synaptic alterations, via downregulation of the miR-7 target, neuroligin 2 [40].

Similarly, HIV Tat-activated microglia-derived EVs (Tat-MEVs) containing the NLRP3 inflammasome were also shown to be taken up by the neurons, resulting in synaptodendritic injury [39]. We have also demonstrated that HIV Tat induces ferroptosis in microglia by increasing Acyl-CoA synthetase long-chain family member 4 (ACSL4), which, in turn, increases the expression of ferritin heavy chain 1 (FTH1) and concomitantly reduces the expression of the antioxidant, GPX4 [43]. Building on these findings, the present study investigates whether HIV Tat-induced MEVs function as intercellular signaling mediators that transfer iron-handling and ferroptosis-associated cargo to neurons, thereby promoting neuronal iron dysregulation and synaptodendritic injury. Specifically, we examined whether Tat-MEVs carrying iron-regulatory proteins contribute to neuronal oxidative stress, mitochondrial dysfunction, and structural and functional synaptic alterations. By exploring a potential transferrin-centered EV-mediated communication pathway between microglia and neurons, this work provides mechanistic insight into how microglial stress responses propagate neurodegenerative signaling in the context of NeuroHIV.

## Materials and methods

### Reagents

Antibodies targeting TF (17435-1-AP, Proteintech Group Inc, IL, USA), TFR1 (ab214039, Abcam, MA, USA), STEAP3 (ab104654, Abcam, MA, USA), DMT1 (ab55735, Abcam, MA, USA), FTH1 (ab75973, Abcam, MA, USA), PSD95 (ab2723, Abcam, MA, USA), GAD65 (ab239372, Abcam, MA, USA), gephyrin (ab181382, Abcam, MA, USA), synaptophysin (17785-1-AP, Proteintech Group Inc, IL, USA), vGlut1 (AB5905, Millipore, Burlington, MA, USA), Alix (ab275377, Abcam, MA, USA), TSG101 (ab125011, Abcam, MA, USA), CD9 (ab92726, Abcam, MA, USA), Calnexin (ab133615, Abcam, MA, USA) were procured from commercial vendors as specified. Reagents used in cell culture included Dulbecco’s Modified Eagle Medium (DMEM, 10-013-CV, NY, USA), heat-inactivated fetal bovine serum (FBS, S11150H, GA, USA, ), penicillin-streptomycin (15140-122, Life Technologies, CA, USA), Hank’s Balanced Salt Solution (HBSS, 14025076, Gibco™, MA, USA), trypsin (25200056, Gibco™, MA, USA), Neurobasal medium (21103049, Invitrogen, MA, USA), L-Glutamate (A2916801, Gibco™, MA, USA), and B27 supplement (17504044, Invitrogen, MA, USA) were purchased. All other chemicals were of analytical grade.

### Cell cultures and treatments

Mouse BV2 microglial cells were used for the isolation of MEVs. The BV2 cell line was generously provided by Dr. Sanjay Maggirwar from the University of Rochester Medical Center, NY, USA. The cells were cultured in DMEM with high glucose, supplemented with 10% heat-inactivated FBS, 100 µg/mL streptomycin, and 100 units/mL penicillin. To prepare for MEV isolation, BV2 cells were seeded at a density of 2 × 10^6^ cells in T150 flasks. The cells were serum-starved overnight, followed by treatment with 100 ng/mL HIV Tat or heat-inactivated HIV Tat (HT) for 48 h in a humidified incubator maintained at 37 °C with 5% CO_2_. For experiments involving iron chelation, BV2 cells were pre-treated with 2 µM deferoxamine (DFO) for 1 h before being treated with 100 ng/mL HIV Tat for 48 h. Conditioned media (30 mL) from each T150 flask were collected and used for MEV isolation.

Rat primary hippocampal (RHN) and rat cortical neurons (RCN) were obtained from embryonic day 18 (E18) rat pups using a previously published protocol [44, 45]. All animal procedures were conducted in accordance with ethical guidelines and approved by the Institutional Animal Care and Use Committee (IACUC) at the University of Nebraska Medical Center. After anesthesia, E18 rat brains were dissected in HBSS buffer. The hippocampi and cortex were carefully isolated and collected in HBSS. After removing the HBSS, 0.25% trypsin was added, and the tissues were incubated in a 37 °C water bath. The tissues were then mechanically dissociated and passed through a 40 μm cell strainer to obtain a single-cell suspension. Hippocampal neurons were seeded at 0.15 × 10^6^ cells per well and cortical neurons at 0.2 × 10^6^ cells per well in poly-D-lysine-coated 24-well plates for western blotting analysis or on coverslips for microscopic imaging. For fluorometric/colorimetric assays or Seahorse assays, neurons were seeded at 0.05 × 10^6^ cells per well in 96-well plates. The cells were maintained in a specialized neuronal culture medium consisting of Neurobasal medium supplemented with penicillin-streptomycin, L-Glutamine, and B27. The culture medium was refreshed every other day by removing half of the medium from each well and replacing it with an equal volume of fresh medium. All experimental treatments were performed directly in the culture medium. The purity of neuronal cultures at DIV21 was assessed by performing triple immunofluorescence staining for MAP2 (neuronal marker), IBA1 (microglia marker), and GFAP (astrocyte marker). Cultures were fixed, permeabilized, and incubated with primary antibodies against the respective markers, followed by appropriate fluorescently labeled Alexa Fluor secondary antibodies. Nuclei were counterstained with DAPI, and images were acquired using a ZEISS ELYRA PS.1 confocal microscope at 20× magnification.

### Isolation of MEVs and non-EV fraction from conditioned media

MEVs were isolated from the conditioned media of untreated BV2 cells (control) or from cells treated with Tat, HT, or DFO using a protocol adapted from previous studies [39, 46]. BV2 cells were first cultured in a serum-free medium for 12 h, followed by 48 h of treatment with 100 ng/mL HIV Tat in the presence or absence of DFO (2µM). Collected conditioned media from treated cultures were then subjected to sequential centrifugation at 300 x *g* and 2000 x *g* for 10 min each to remove cell debris. The supernatant was further centrifuged at 10,000 x *g* for 30 min. The resulting supernatant was filtered through 0.8 μm and 0.22 μm filters, and the filtrate was ultracentrifuged at 100,000 x *g* for 70 min at 4 °C using a Beckman Ti32 rotor (Brea, CA, USA). The final EV pellet was resuspended in 300 µL of PBS. To control for potential soluble carryover effects of residual Tat or DFO during EV isolation, the culture supernatants remaining post-ultracentrifugation were retained as the corresponding non-EV fraction, representing the soluble component source remaining after EV removal. The isolated EVs were characterized by nanoparticle tracking analysis (NTA, ZetaView NS300, Particle Metrix, Germany), and exosome-specific markers were validated via western blotting. For neuronal treatment experiments, RHNs and RCNs were treated with MEVs at a concentration of 500 MEVs/cell for 48 h. In parallel experiments, neurons were also treated with the corresponding non-EV fractions derived from the same conditioned media, for 48 h.

### Nanoparticle tracking analysis

The size distribution and zeta potential of the isolated MEVs were analyzed using a ZetaView nanoparticle tracking system (ZetaView NS300, Particle Metrix, Germany) operated with ZetaView 8.04.02 SP1 software. MEVs were diluted 1:1000 in PBS, injected into the system, and analyzed with a sensitivity setting of 85, a frame rate of 30, and two readings per position.

### Transmission electron microscopy (TEM)

For ultrastructural analysis, RHN were seeded onto coverslips in 6-well plates at a density of 2 × 10^6^ cells per well and treated with MEVs for 48 h. After treatment, cells were washed with PBS and fixed in a solution containing 2% glutaraldehyde, 2% paraformaldehyde, and 0.1 M cacodylate buffer for 30 min at room temperature. The samples were then stored at 4 °C until they were processed for TEM analysis. The ultrastructure was observed using an FEI Tecnai G2 Spirit transmission electron microscope (FEI, Houston, TX, USA).

### Western blotting

After the specified treatments, neurons (RHN, and RCN) were harvested using RIPA lysis buffer.

The total protein (10 µg) from each lysate was separated by sodium dodecyl sulfate-polyacrylamide gel electrophoresis (SDS-PAGE) and transferred to polyvinylidene difluoride (PVDF) membranes (Millipore IPVH00010). For EV samples, equal numbers of EVs per condition, as determined by nanoparticle tracking analysis (NTA), were loaded for analysis of both canonical EV markers and iron-related cargo proteins. Membranes were blocked in 5% milk prepared in 1x tris-buffered saline with tween 20 solution (TBST) buffer, washed three times with 1x TBST, and incubated overnight with primary antibodies specific to the proteins of interest. The membranes were then washed and incubated with horseradish peroxidase (HRP)-conjugated secondary antibodies (anti-mouse or anti-rabbit IgG) to detect the proteins of interest.

β-actin (A5441, Millipore Sigma, MA, USA) was used as an internal control for neuronal lysates, and Calnexin was included as negative control for EV samples to confirm absence of cellular contamination.

### qPCR quantification of EV biogenesis markers

The qPCR analyses were conducted in accordance with previously established methodologies [28478506, 29966509]. Total RNA was extracted using the Quick-RNA™ MiniPrep Plus kit (Cat. No. R1058, Zymo Research, Irvine, CA, USA) following the manufacturer’s instructions.

Reverse transcription of purified RNA was performed using the Verso cDNA Synthesis Kit for qPCR (Cat. No. AB-1453/B, Thermo Fisher Scientific, Pittsburgh, PA, USA). Gene expression levels of the EV biogenesis markers smpd3 (Forward primer, GCTGCTGGACACATACCCAT; Reverse primer GCAACTCTGACCGTGGATGT), pdcd6ip (Forward primer, GCTAAGCTGGCAAATCAGGC; Reverse primer, GGGGAATACCTCCTTGGGGA), tsg101 (Forward primer, GCTGCTGGACACATACCCAT; Reverse primer GCAACTCTGACCGTGGATGT), and rab27a (Forward primer, GAGCACCAAACCGGGTGA; Reverse primer, TACCCCAGAGTCTCCCAAGG) were quantified using SYBR^®^ Green Quantitative RT-qPCR Mastermix (Cat. No. 330513, Qiagen, Germantown, MD, USA).

Amplification and fluorescence detection were carried out on an Applied Biosystems^®^ QuantStudio™ 3 Real-Time PCR System. All reactions were run in technical triplicates using six independent biological replicates. Target gene expression was normalized to the housekeeping genes gapdh, and relative expression levels were calculated using the 2⁻ΔΔCt method.

### ExoSparkler tagging of MEVs

MEVs were labeled using the ExoSparkler Exosome Membrane Labeling Kit-Red (EX02, Dojindo, MD, USA) according to the manufacturer’s instructions, with PBS processed in parallel as a labeling control.

### Lactate dehydrogenase assay

LDH release, an indicator of cell membrane damage, was measured in RHNs, and RCNs using the LDH Assay Kit (ab65393, Abcam, Boston, MA, USA). Cells were seeded in 96-well plates at a density of 5 × 10^4^ cells per well and treated with Con MEVs, Tat-MEVs, DFO + Tat MEVs, and DFO MEVs. Supernatants were collected post-centrifugation at 600 x g for 10 min and analyzed for LDH activity according to the manufacturer’s instructions. Absorbance at 450 nm was measured using a Synergy MX Multi-Mode Microplate Reader (BioTek Instruments, Winooski, VT, USA). Each condition was tested in triplicates.

### Lysosomal membrane permeability assay

RHNs were seeded in 96-well plates at 0.05 × 10^6^ cells per well and treated with MEVs for 48 h. After incubation, the media was removed, and acridine orange (158850, Millipore Sigma, MA, USA) was added at a concentration of 5 µg/mL, followed by a 15-minute incubation at 37 °C.

Fluorescence intensity was measured using a Synergy™ Mx Monochromator-Based Multi-Mode Microplate Reader (BioTek Instruments, Inc., Winooski, VT, USA) with excitation at 485 nm and emission recorded at 530 nm and 620 nm, which was then used to calculate the lysosomal membrane permeability (LMP).

### Lysosomal pH measurement

RHNs were seeded at a density of 0.15 × 10^6^ cells per well on coverslips in a 24-well plate and transfected overnight with a pFUGW-FIRE-pHLy plasmid (170774, Addgene, MA, USA) using Opti-MEM™ medium and Lipofectamine 3000 reagent, as per the manufacturer’s instructions, to measure lysosomal pH levels. After transfection, the medium was replaced with fresh growth medium, and the cells were treated with MEVs for 48 h. Following treatment, the cells were washed with PBS, and the coverslips were mounted on glass slides with DAPI. Fluorescence images were captured using a fluorescence microscope (BioTek Instruments, Inc., VT, USA) with excitation wavelengths of 488 nm (monomeric teal fluorescent protein 1 [mTFP1]) and 594 nm (mCherry). The mCherry fluorescence intensity, representing the pH-insensitive cytosolic domain of the reporter, remained constant across all groups. In contrast, the mTFP1 fluorescence intensity, which is pH-sensitive, varied, reflecting changes in lysosomal pH. The mTFP1/mCherry ratio was calculated and compared to a standard curve to determine lysosomal pH values, as described in a previous publication [47].

### Intracellular iron quantification

The RHNs were cultured in a 24-well plate and treated with MEVs for 48 h, as previously described [39]. Subsequently, the labile iron pool (LIP) was quantified using an iron assay kit (ab83366, Abcam, MA, USA) according to the manufacturer’s instructions.

### Endolysosomal iron quantification

To measure endolysosomal iron levels, RHNs were seeded at 0.15 × 10^6^ cells per well on coverslips and transfected with a Lamp1-GFP plasmid (16290, Addgene, MA, USA) for 6 h using OptiMEM media and Lipofectamine 2000. After 6 h, the medium was replaced with a fresh neuronal growth medium, and the cells were incubated for an additional 4 days. Cells were then treated with MEVs for 48 h. Fe^2+^ levels were visualized using FeRhoNox-1 (30 µM; BioTracker 575 Red Fe^2+^ Dye, SCT030, Merck Millipore, MA, USA), a specific probe for Fe^2+^, incubated at 37 °C for 1 h [48]. Cells were washed with PBS, and fluorescence images were acquired on a Zeiss LSM800 confocal microscope with excitation at 537 nm and emission at 569 nm. Z-stack images were acquired at 0.4 μm intervals. Fluorescence intensity was quantified as mean fluorescence intensity (MFI) using ZEN Blue software (v3.6), with image acquisition and analysis performed blind to treatment. Data are presented as mean fluorescence intensity measured across 6 independent image fields from three biological replicates, with 2 technical replicates per biological replicate. Each field contained an average of 5–6 neurons.

### Cytosolic iron quantification

For cytosolic iron measurement, RHNs were seeded at 0.15 × 10^6^ cells per well on coverslips and transfected with a Lamp1-RFP plasmid (1817, Addgene, MA, USA) for 6 h using Opti-MEM™ media and Lipofectamine 2000. Following the transfection, cells were incubated in fresh growth media for 4 days. Afterward, the cells were treated with MEVs for 48 h. Cytosolic Fe^2+^ was measured using PhenGreen™ SK diacetate (P14313, Thermo Fisher Scientific, MA, USA), a probe that quenches upon binding to cytosolic iron. The cells were incubated with 10 µM PhenGreen™ SK diacetate at 37 °C for 30 min [49]. Cells were washed with PBS, and images were captured on a Zeiss LSM800 confocal microscope with excitation at 537 nm and emission at 569 nm. Z-stacks were acquired at 0.4 μm intervals. Analysis was performed using ZEN Blue software. Data are presented as mean fluorescence intensity measured across 6 independent image fields from three biological replicates, with 2 technical replicates per biological replicate. Each field contained an average of 5–6 neurons.

### Cytosolic ROS measurement

Cytosolic ROS levels were determined using the Image-IT™ Live Green ROS detection kit (I36007, Invitrogen, MA, USA), according to the manufacturer’s protocol. RHNs were seeded onto coverslips in 24-well plates and treated with MEVs for 48 h. After treatment, cells were washed with HBSS and incubated with 25 mM carboxy-H2DCFDA, a ROS-sensitive dye, at 37 °C for 30 min. The cells were then washed with HBSS and mounted using a DAPI-containing mounting medium (36935, Invitrogen, MA, USA). Fluorescent images were captured using a Zeiss Observer Z1 inverted microscope (Carl Zeiss MicroImaging), and images were processed using AxioVs 40 software (version 4.8.0.0, Carl Zeiss MicroImaging).

### Mitochondrial ROS measurement

The generation of mitochondrial superoxide radicals was measured using MitoSOX™ Red dye (M36008, Invitrogen, MA, USA), which selectively targets mitochondria and fluoresces upon oxidation by superoxide. RHNs were seeded onto coverslips in 24-well plates at a density of 0.15 × 10^6^ cells per well and treated with MEVs for 48 h. Following the treatment, cells were washed with PBS and incubated with 5 µM MitoSOX™ Red dye for 15 min at 37 °C. After staining, cells were washed and imaged using a Zeiss Observer Z1 inverted microscope. To minimize nuclear accumulation of the dye, images were taken within 10–20 min after staining, as the dye can accumulate in the nucleus after approximately 40 min.

### Mitochondrial functional analysis

Mitochondrial function was assessed by measuring the oxygen consumption rate (OCR) and extracellular acidification rate (ECAR) using the Seahorse XF Cell Mito Stress Test Kit (103015-100, Agilent, CA, USA), and Seahorse XFp Extracellular Flux Analyzer (Seahorse Bioscience, Billerica, MA, USA). RHNs were seeded at a density of 0.05 × 10^6^ cells per well in Seahorse XFe96 cell culture plates, followed by treatment with MEVs for 48 h at 37 °C with 5% CO_2_.

Prior to the assay, a “Flux Pak” cartridge containing O_2_^−^ and H^+^-sensitive fluorophores was hydrated in XF Calibrant solution and incubated in a non-CO_2_ incubator overnight at 37 °C. On the day of the assay, the culture media was replaced with XF assay medium (bicarbonate-free DMEM supplemented with 10 mM glucose, 2 mM pyruvate, and 2 mM L-glutamine), and the cells were incubated in a non-CO_2_ incubator for degassing. Mitochondrial complex inhibitors were loaded into the Flux Pak, and OCR and ECAR values were recorded using the Seahorse MitoStress Test protocol. Data were analyzed using the Seahorse Wave 2.2.0 software (Seahorse Bioscience).

### GFP plasmid transfection and immunocytochemistry

GFP plasmid transfection was performed on RHNs seeded at a density of 0.15 × 10^6^ cells per well using Lipofectamine 2000 according to the manufacturer’s instructions. Neurons were transfected with 500 ng of GFP-expressing plasmid (13031, Addgene, MA, USA) mixed with 1 µL of Lipofectamine 2000, diluted in 25 µL of Opti-MEM media. After 6 h of transfection, the medium was replaced with fresh growth media, and the cells were incubated for an additional 96 h. Post-transfection, neurons were treated with MEVs for 48 h. Cells were then fixed with 4% paraformaldehyde for 20 min at room temperature and permeabilized using 0.1% Triton X-100. After blocking with 5% normal goat serum in PBS for 2 h, primary antibodies against gephyrin and vGlut1 were added and incubated overnight. Secondary antibodies conjugated to Alexa Fluor 594 were then applied, followed by mounting with Prolong Gold antifade reagent with DAPI. Confocal z-stack images were acquired using a ZEISS LSM800 confocal microscope at 63× magnification. For Sholl analysis and dendritic spine quantification, Neurolucida 360 neuronal reconstruction software (version 2021.1.1) was used. Only mature neurons with clearly defined dendritic arbors were included for analysis. Secondary and tertiary dendritic segments were selected for quantification, and multiple spines per dendritic segment were traced. Data were collected from 6 independent neuronal cultures, with 1–2 neurons analyzed per biological replicate, and each neuron included all observable dendritic branches in the analysis. Image acquisition and analysis were performed blind to the treatment group to reduce bias.

### Electrophysiology

Whole-cell electrophysiology was performed on RHNs (DIV 19–21) using established protocols [40]. Neurons were seeded on coverslips, and signals were filtered at 2 kHz and digitized at 10 kHz using an Axon Digidata 1440 A analog-to-digital board (Molecular Devices, San Jose, CA, USA).

Recordings were accepted if pipette access resistance was less than 20 MΩ and remained stable during the recording. The external solution used contained 150 mM NaCl, 3 mM KCl, 10 mM HEPES, 6 mM mannitol, 0.02 mM EDTA, 1.5 mM MgCl_2_, and 2.5 mM CaCl_2_ at pH 7.4. Glass pipettes with a resistance of 2–5 MΩ were filled with an internal solution composed of 110 mM cesium gluconate, 30 mM CsCl, 5 mM HEPES, 4 mM NaCl, 0.5 mM CaCl_2_, 2 mM MgCl_2_, 5 mM BAPTA, 2 mM Na_2_ATP, and 0.3 mM Na_2_GTP at pH 7.35. QX-314 was added to block voltage-gated sodium channels. Miniature excitatory postsynaptic currents (mEPSCs) were recorded in the presence of 0.5 µM tetrodotoxin and 100 µM picrotoxin at a holding potential of − 70 mV. Analysis of mEPSCs was conducted using Minianalysis software (Synaptosoft, Atlanta, GA, USA), with an amplitude threshold of 5 pA and measurement of miniature current frequency. Cells from 6 independent neuronal cultures, each treated with EVs derived from six biological replicates of BV2 cells were analyzed. A total of 10–15 neurons were recorded and analyzed across all replicates (2–3 neurons per biological replicate).

### Statistical analysis

All statistical analyses were performed using GraphPad Prism (version 10.1, GraphPad Software, San Diego, CA, USA). Data are presented as the mean ± standard error of the mean (SEM) from at least six independent experiments unless otherwise stated. For comparisons between the two groups, an unpaired two-tailed Student’s t-test was applied. For comparisons involving more than two groups, one-way analysis of variance (ANOVA) was used, followed by Tukey’s post-hoc tests to determine specific group differences. Statistical significance was set at *p* < 0.05. Graphs and visual data representations were created using GraphPad Prism.

## Results

### HIV Tat MEVs (Tat-MEVs) induce neuronal dysregulation and iron-dependent neurotoxicity in rat hippocampal and cortical neurons

To investigate whether HIV Tat activated iron-dependent ferroptotic signaling in microglia, BV2 cells were treated with HIV Tat (100 ng/mL) or heat-inactivated Tat (HT) for 48 h, and the expression of key iron-handling proteins associated with ferroptosis signaling, transferrin (TF), transferrin receptor 1 (TFR1), STEAP3, divalent metal transporter 1 (DMT1), and ferritin heavy chain 1 (FTH1) was assessed. HIV Tat treatment markedly increased the expression of all the iron-regulatory proteins compared with control or HT-treated cells (Supplementary Fig. 1A-E). To further establish the contribution of iron to this response, BV2 cells were pretreated with the iron chelator deferoxamine (DFO; 2 µM, 1 h) prior to HIV Tat exposure. DFO pretreatment attenuated the HIV Tat-induced upregulation of iron-regulatory proteins (Supplementary Fig. 1F-J), confirming that HIV Tat promotes activation of an iron-dependent pathways associated with ferroptotic signaling in microglial cells. To extend these findings, we next examined whether DFO pretreatment altered EV biogenesis pathways in BV2 cells. RT-qPCR analysis of key EV biogenesis-associated genes, including smpd3, tsg101, pdcd6ip, and rab27a, revealed no significant changes in transcript levels in DFO-pretreated BV2 cells compared with control cells (Supplementary Fig. 1K-N).

Further, MEVs were isolated from the conditioned media of BV2 cells treated with HIV Tat (100 ng/ml) or heated HIV Tat (HT) for 48 h, and their purity was confirmed as described in our previous studies (Supplementary Fig. 2A) [39], including detailed EV characterization by NTA, TEM. and EV markers. MEVs isolated from the conditioned media of BV2 cells exposed to heat-inactivated Tat (HT MEVs) were used as negative controls. The total number and size distribution of EVs were assessed using NTA. BV2 cells exposed to HIV Tat (100 ng/mL for 48 h) exhibited increased release of MEVs compared to control or HT-treated cells and ranged in size from 50 to 200 nm (Supplementary Fig. 2B-C). Western blot analysis confirmed the expression of EV markers (Alix, TSG101, and CD9) and the absence of calnexin in the MEVs, indicating the purity of isolated MEVs (Supplementary Fig. 2D). We also analyzed the expression of iron-handling proteins, TF, TFR1 and FTH1 in the Tat-MEVs and found that HIV Tat exposure upregulated the expression and release of these proteins in Tat-MEVs isolated from BV2 cells (Supplementary Fig. 2E).

**Fig. 1:**
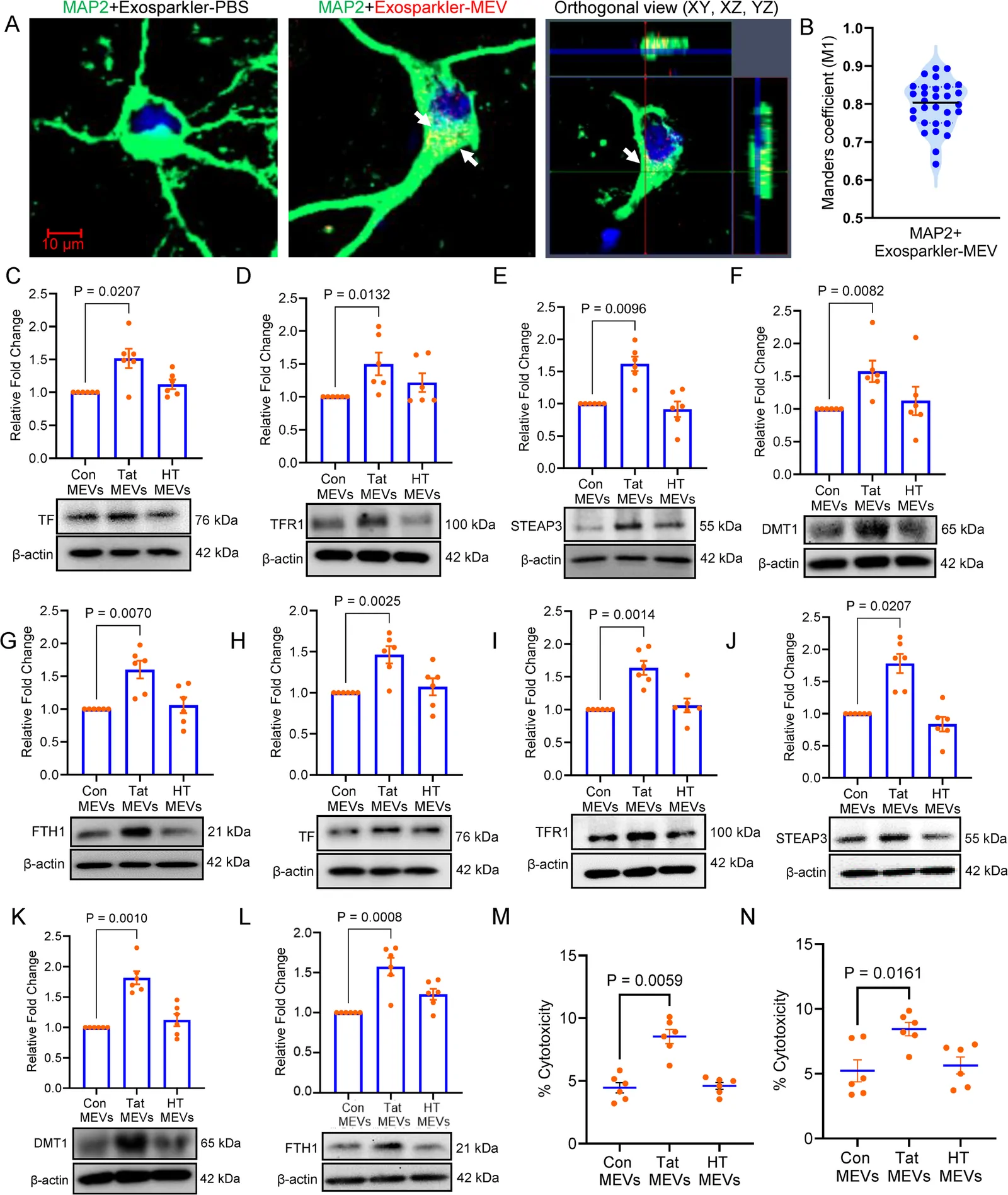
Effect of MEVs on iron-regulatory proteins: (**A**) Representative merged confocal images showing Exosparkler-labeled PBS control (Exo-PBS) in and MAP2-positive neurons (green); ExoSparkler-labeled MEVs (Exo-MEVs) (red) in MAP2-positive neurons (green); and Orthogonal (XY, XZ, and YZ) views are shown to confirm 3D co-localization of Exo-MEV signal within neuronal structures. **B** Quantification of MEV-neuron co-localization expressed as Manders coefficient (M1), calculated using the ZEN Blue colocalization tool. Data are presented as mean ± SEM from *n* = 15 cells analyzed across 3 independent experiments. Representative western blots showing the expression of iron regulatory proteins (**C**) TF, (**D**) TFR1, (**E**) STEAP3, (**F**) DMT1 and (**G**) FTH1 in Con MEVs, Tat MEVs, and HT MEVs-treated RHNs and (**H**) TF, (**I**) TFR1, (**J**) STEAP3, (**K**) DMT1 and (**L**) FTH1 in Con MEVs, Tat MEVs, and HT MEVs-treated RCNs. Representative graphs showing cytotoxicity in neurons following treatment with Con MEVs, Tat MEVs, and HT MEVs in (**M**) RHNs and (**N**) RCNs which were assessed for LDH release, and cytotoxicity was expressed as % cytotoxicity. Data are presented as mean ± SEM from *n* = 6 biological independent experiments. One-way ANOVA followed by Kruskal-Wallis post hoc test was performed. *P* < 0.05 is considered statistical significance between treatment groups. DMT1, divalent metal transporter 1; FTH1, ferritin heavy chain 1; MEV, microglia-derived EVs; RCN, rat cortical neurons; RHN, rat hippocampal neurons; STEAP3, six-transmembrane epithelial antigen of prostate 3; TF, transferrin; TFR1, transferrin receptor 1

To investigate the impact of iron-mediators cargo-containing MEVs on neuronal morphology, we examined their uptake by primary rat hippocampal (RHNs) and cortical neurons (RCNs. MEVs were labeled with the EV-specific membrane dye ExoSparkler and applied to neurons which were later stained with MAP2 (neuronal marker). Both RHNs and RCNs efficiently internalized the labeled MEVs, as demonstrated by clear colocalization of ExoSparkler red fluorescence with MAP2-positive neurons- green fluorescence (Fig. 1A). Quantitative analysis of colocalization using the Manders coefficient revealed values ranging from 0.65 to 0.88, indicating substantial overlap and confirming effective MEV uptake by neuronal populations (Fig. 1B). We then sought to examine the expression levels of transferrin-regulated iron-handling proteins, including TF, TFR1, STEAP3, and DMT1, in RHNs and RCNs (days in vitro 21) exposed to either control MEVs (Con MEVs) or Tat-MEVs at a concentration of 500 MEVs/cell (based on our previous report) [39]. As shown in Fig. 1C-G, RHNs exposed to Tat MEVs demonstrated significantly upregulated expression of TF, TFR1, STEAP3, DMT1, and FTH1 compared to RHNs exposed to either Con MEVs or HT MEVs. Similar results were observed for RCNs (Fig. 1H-L).

Simultaneously, to determine whether the internalized Tat-MEVs induced neuronal injury, we performed an LDH cytotoxicity assay on RHNs, and RCNs exposed to Con MEVs, Tat MEVs, or HT MEVs. As shown in Fig. 1M (RHNs), and Fig. 1N (RCNs), neurons treated with Tat-MEVs exhibited a significant increase in % cytotoxicity compared with those exposed to Con MEVs, indicating that Tat-MEVs induced measurable neuronal toxicity.

To further explore the role of the iron-dependent signaling pathways in BV2 cells and their downstream effects on neurons, BV2 cells were pre-treated with the iron chelator, deferoxamine (DFO; 2 µM) for 1 h, followed by exposure of cells to HIV Tat (100 ng/ml) for 48 h.

Conditioned media was subsequently collected, and EVs isolated as described earlier [46]. Consistent with our earlier observations, Tat-treated BV2 cells exhibited an increased release of MEVs compared with control cells. However, DFO pretreatment reduced the overall EV number relative to Tat treatment alone (Supplementary Fig. 2F). To confirm that changes in EV cargo were specific and not due to global alterations in EV content or contamination, we assessed canonical EV markers TSG101 and Alix, as well as Calnexin as a negative control for nuclear and cellular contamination. Levels of TSG101 and Alix remained consistent across all EV conditions, while Calnexin was undetectable in all EV preparations, confirming the purity of the EV isolates (Supplementary Fig. 2G). Importantly, analysis of iron-regulatory proteins revealed that TF, TFR1, and FTH1 were markedly elevated in Tat-MEV cargo, whereas their levels were substantially reduced in DFO + Tat MEVs and DFO MEVs (Supplementary Fig. 2H).

**Fig. 2:**
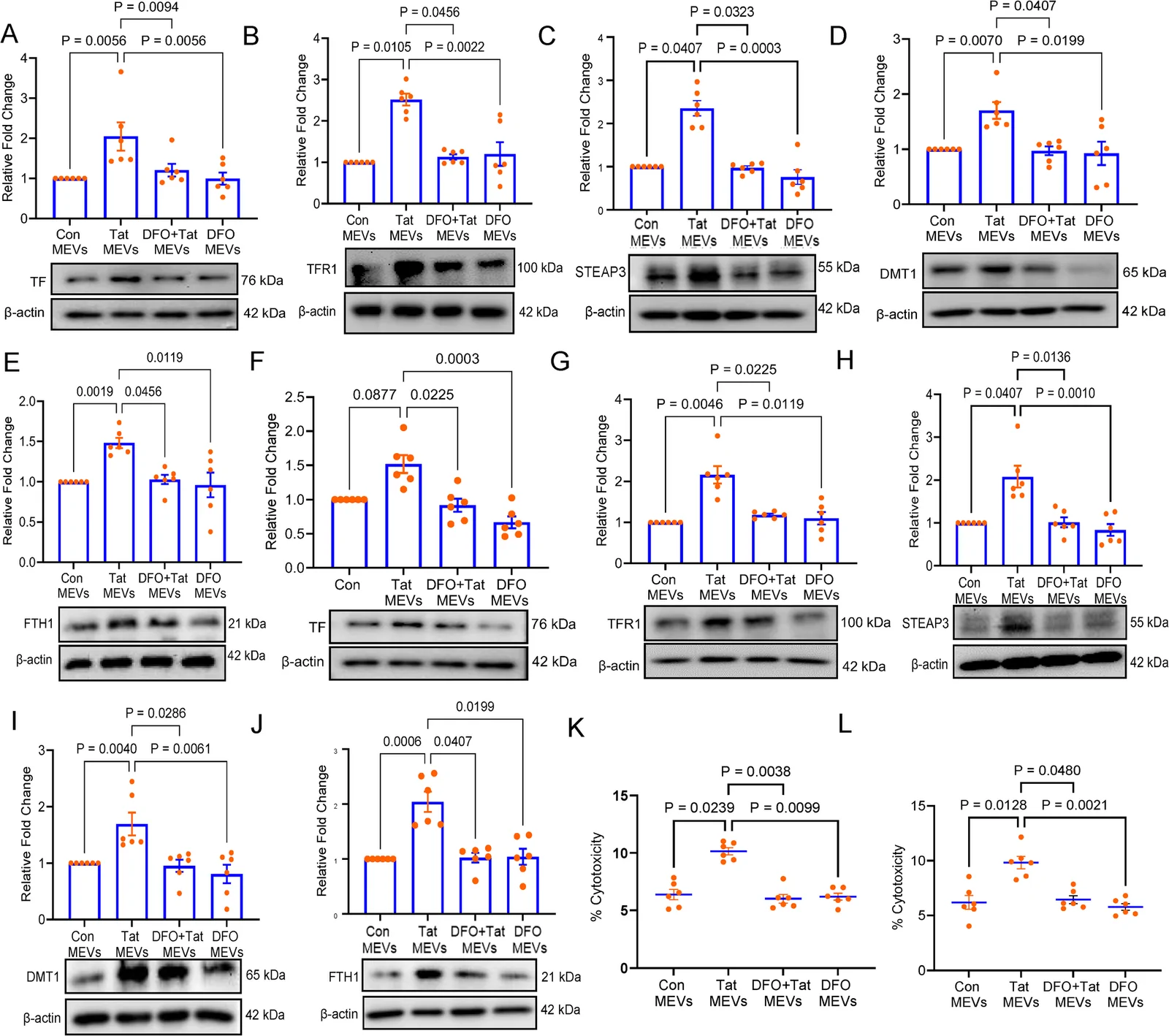
Effect of DFO MEVs on iron-regulatory proteins: Representative western blots showing the expression of (**A**) TF, (**B**) TFR1, (**C**) STEAP3, (**D**) DMT1 and (**E**) FTH1 in Con MEVs, Tat MEVs, DFO + Tat MEVs, and DFO MEVs-treated RHNs and (**F**) TF, (**G**) TFR1, (**H**) STEAP3, (**I**) DMT1 and (**J**) FTH1 in Con MEVs, Tat MEVs, DFO + Tat MEVs, and DFO MEVs-treated RCNs. Representative graphs showing cytotoxicity in neurons following treatment with Con MEVs, Tat MEVs, DFO + Tat MEVs, and DFO MEVs in (**K**) RHNs and (**L**) RCNs which were assessed for LDH release, and cytotoxicity was expressed as % cytotoxicity. Data are presented as mean ± SEM from *n* = 6 biological independent experiments. One-way ANOVA followed by Kruskal-Wallis post hoc test was performed. *P* < 0.05 is considered statistical significance between treatment groups. DFO, deferoxamine; DMT1, divalent metal transporter 1; FTH1, ferritin heavy chain 1; MEV, microglia-derived EVs; RCN, rat cortical neurons; RHN, rat hippocampal neurons; STEAP3, six-transmembrane epithelial antigen of prostate 3; TF, transferrin; TFR1, transferrin receptor 1

RHNs and RCNs (days in vitro 21) were then exposed to either Con MEVs, Tat-MEVs, DFO + Tat-MEVs, or DFO-MEVs at a concentration of 500 MEVs/cell for 48 h. As shown in Fig. 2A and J, Tat-MEVs significantly upregulated the expression of TF, TFR1, STEAP3, DMT1, and FTH1, in both RHNs and RCNs, while exposure to either DFO + Tat MEVs or DFO MEVs downregulated the expression of these proteins in both RHNs (Fig. 2A and E) and RCNs (Fig. 2F and J). Additionally, to distinguish EV-mediated effects from potential soluble carryover effects of either DFO or Tat, both RHNs and RCNs were also treated with the corresponding non-EV fractions obtained during the EV isolation procedure for each condition and assessed for expression of TF and FTH1 proteins. We observed increased expression of both TF and FTH1 in RHNs (Supplementary Fig. 3A-B), as well as RCNs (Supplementary Fig. 3C-D) exposed to Tat-MEVs compared with the neurons treated with Con-MEVs. Notably, treatment with the corresponding non-EV fractions did not alter the expression of either TF or FTH1, with their levels comparable to those observed in Con-MEV treated neurons.

To further evaluate the contribution of EV-mediated transfer of iron-handling proteins to the neuronal responses observed following Tat exposure BV2 cells were pre-treated with the EV release inhibitor GW4869 (2 µM) for 1 h, followed by exposure of the cells to HIV Tat (100 ng/mL). Conditioned media was collected at 48 h, and MEVs were isolated as described earlier.

Pre-treatment of BV2 cells with GW4869 inhibited the release of EVs as expected (Supplementary Fig. 4A), with a concomitantly downregulated expression of the TF, TFR1, & FTH1 in the Tat-MEV cargo (Supplementary Fig. 4B). Next, RHNs and RCNs were exposed to Con MEVs, Tat MEVs, GW4869 + Tat MEVs (GW + Tat MEVs), or GW4869 MEVs (GW MEVs), followed by assessment of the expression of iron-handling proteins in neurons. Exposure of RHNs and RCNs to Tat MEVs significantly upregulated the expression of TF, TFR1, STEAP3, DMT1, and FTH1 (RHNs: Supplementary Fig. 4C-G) and (RCNs: Supplementary Fig. 4H-L). In contrast and, as expected, exposure of RHNs, and RCNs to GW + Tat MEVs or GW MEVs failed to upregulate the expression of these proteins (Supplementary Fig. 4C-L).

Although these findings demonstrate neuronal cytotoxicity following Tat-MEV exposure, the assays performed in the present study do not distinguish ferroptotic cell death from other forms of neuronal injury. 

### Tat MEVs increased the endolysosomal pH, decreased endolysosomal iron, and increased cytosolic iron in rat hippocampal neurons.

**Fig. 3:**
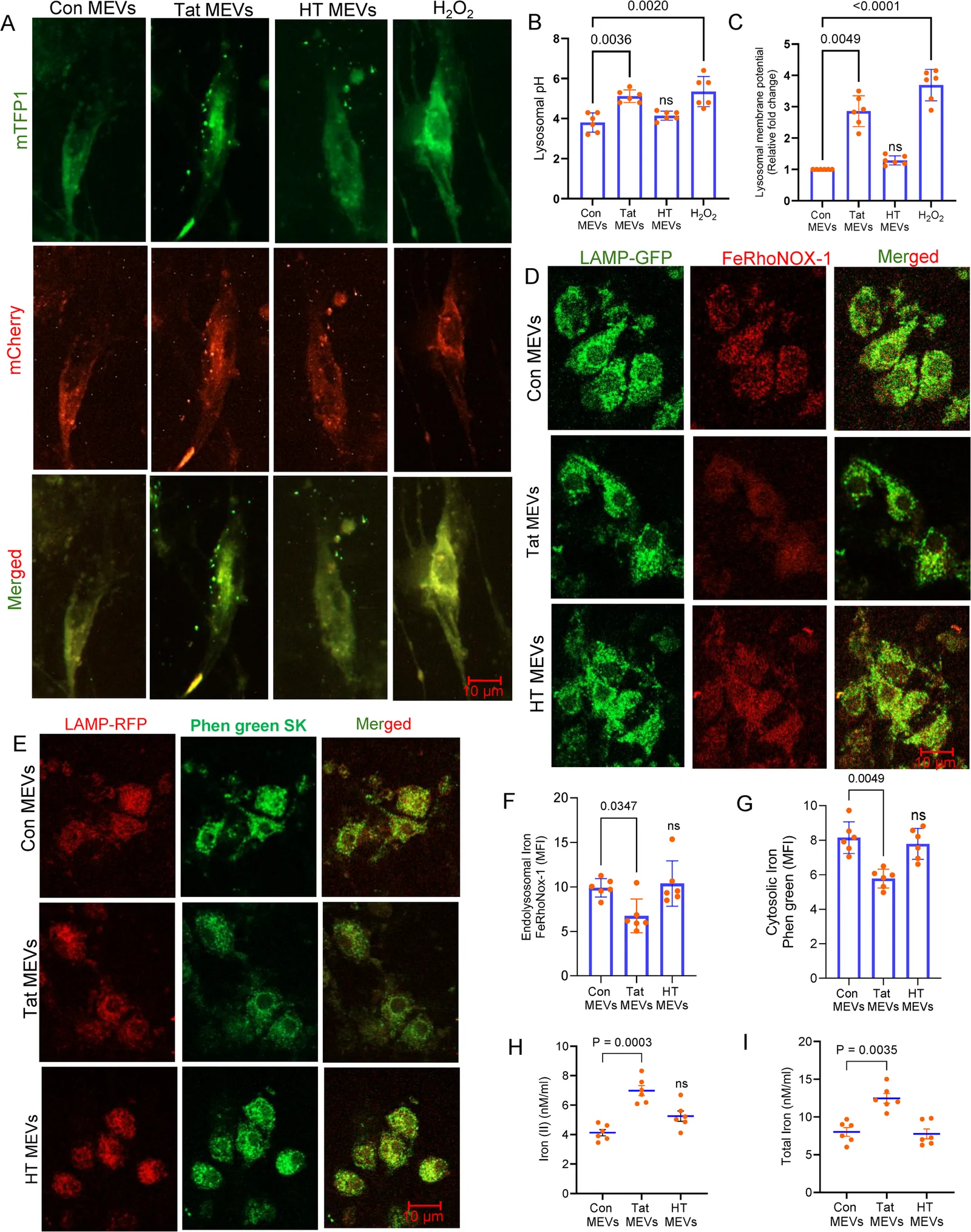
Role of MEVs in endolysosomal iron efflux: Representative images, and quantification of (**A**, **B**) lysosomal pH; (**C**) lysosomal membrane potential; (**D**, **F**) endolysosomal iron content, (**E**, **G**) cytosolic iron content in RHN transfected with pFUGW-FIRE-pHLy, LAMP-GFP, and LAMP-RFP plasmids, respectively, followed by treatment with Con MEVs, Tat MEVs, and HT MEVs. (**H**, **I**) Quantification of intracellular iron content by measuring the labile iron pool in RHNs treated with Con MEVs, Tat MEVs, and HT MEVs. Data are presented as mean fluorescence intensity measured across 6 independent image fields from three biological replicates, with 2 technical replicates per biological replicate. Each field contained an average of 5–6 neurons. One-way ANOVA followed by Kruskal-Wallis post hoc test was performed. *P* < 0.05 is considered statistical significance between treatment groups. HT, heat-inactivated Tat; MEV, microglia-derived EVs; RHN, rat hippocampal neurons

Given that HIV Tat has been reported to induce endolysosomal deacidification by disrupting membrane potential in neurons, which is associated with iron, we next evaluated the effects of Tat-MEVs on endolysosomal pH and lysosomal membrane potential. For this, we resorted to RHNs, which were treated with either Con MEVs, Tat MEVs, or HT MEVs for 48 h, followed by incubation with acridine orange to assess the lysosomal membrane permeability. We also transfected the RHNs with pFUGW-FIRE-pHLy plasmid, followed by treatment with Con MEVs, Tat MEVs, or HT MEVs for 48 h to evaluate the lysosomal pH. As shown in Fig. 3A-C, RHNs exposed to Tat MEVs demonstrated increased endolysosomal pH and lysosomal membrane permeability, similar to those cells treated with hydrogen peroxide (positive control).

**Fig. 4:**
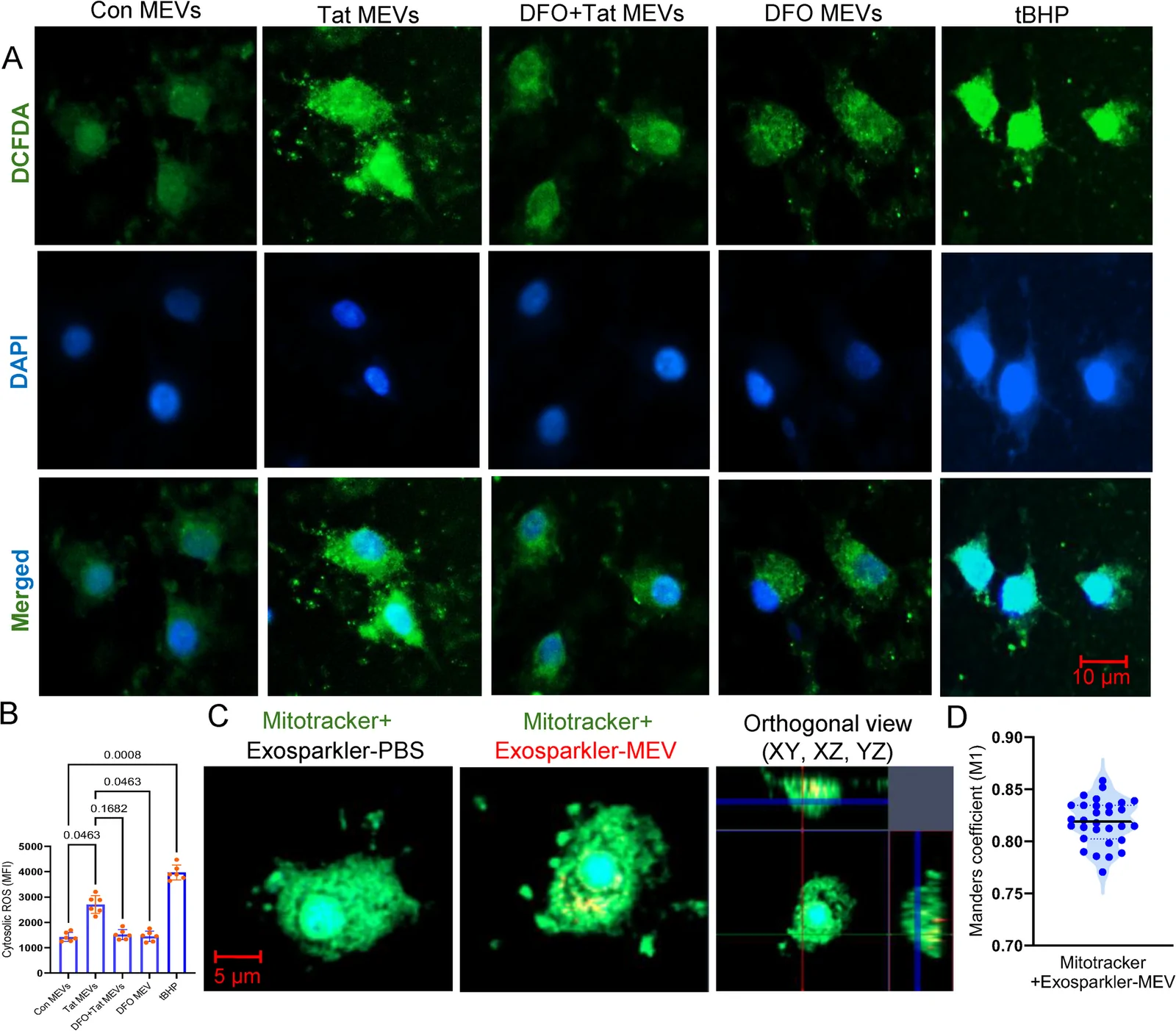
Effect of MEVs on cytosolic ROS: Representative images (**A**), and quantification of (**B**) cytosolic ROS in RHNs treated with Con MEVs, Tat MEVs, DFO + Tat MEVs, and DFO MEVs. Data are presented as mean ± SEM from 3 independent neuronal cultures, each treated with MEVs derived from 3 biological replicates of BV2 cells. A total of 6 ROI fields were analyzed across all replicates (2–3 neurons per field of biological replicates). **C** Representative merged confocal images showing Exosparkler-labeled PBS control (Exo-PBS) in and Mitotracker-stained neurons (green); ExoSparkler-labeled MEVs (Exo-MEVs) (red) in Mitotracker-stained neuron (green); and Orthogonal (XY, XZ, and YZ) views are shown to confirm 3D co-localization of Exo-MEV signal within neuronal structures. **D** Quantification of MEV-neuron co-localization expressed as Manders coefficient (M1), calculated using the ZEN Blue colocalization tool. Data are presented as mean ± SEM from *n* = 15 cells analyzed across 3 independent experiments. *P* < 0.05 is considered statistical significance between treatment groups. DFO, deferoxamine; HT, heat-inactivated Tat; MEV, microglia-derived EVs; RHN, rat hippocampal neurons; ROI, region of interest; ROS, reactive oxygen species

RHNs exposed to HT MEVs, on the other hand, failed to demonstrate any noticeable change in either the endolysosomal pH or lysosomal membrane permeability, an effect similar to RHNs exposed to control MEVs. Since the deacidification of endolysosomes is known to trigger the release of divalent metal cations, such as iron and calcium, from endolysosomes into the cytosol [22, 23], we next investigated the effect of Tat-MEVs on endolysosomal iron levels. For this, RHNs were transfected with LAMP-GFP plasmid and exposed to either Con MEVs, Tat MEVs, or HT MEVs for 48 h, followed by staining with FeRhoNox-1, a Fe²⁺-specific dye [22]. As depicted in Fig. 3D and F, Tat MEVs significantly reduced the mean fluorescence intensity (MFI) in RHNs, indicating a decrease in iron levels within the endolysosomes. Next, we evaluated whether the reduction in endosomal iron levels was accompanied by an increase in cytosolic iron in these cells. For this, RHNs were transfected with LAMP-RFP and treated with Con MEVs, Tat MEVs, or HT MEVs for 48 h, followed by staining the cells with PhenGreen SK, a quenching probe for cytosolic iron [49]. Since PhenGreen SK fluorescence is quenched in the presence of iron, a reduction in MFI indicates increased cytosolic iron. As shown in Fig. 3E, and 3G, RHNs exposed to Tat MEVs demonstrated a significantly reduced MFI compared to the control group, indicating an increase in cytosolic iron in these cells. To further confirm these observations, we also measured intracellular iron content, i.e., labile iron pool (LIP), using an iron assay kit. As shown in Fig. 3H-I, Tat MEV-exposed RHNs demonstrated significantly increased Fe^2+^ iron levels. Since elevated Fe^2+^ levels can generate ROS through Fenton’s reaction, we also assessed the effects of Con MEVs, Tat MEVs, DFO + Tat MEVs, and DFO MEVs on cytosolic ROS levels. As shown in Fig. 4A and B, exposure of RHNs to Tat MEVs resulted in significantly increased ROS generation in the cytosol, an effect that was abrogated in neurons exposed to either DFO MEVs or DFO + Tat MEVs.

### Tat MEVs mediated changes in mitochondrial iron levels, ROS levels, and mitochondrial structure and function in rat hippocampal neurons.

**Fig. 5:**
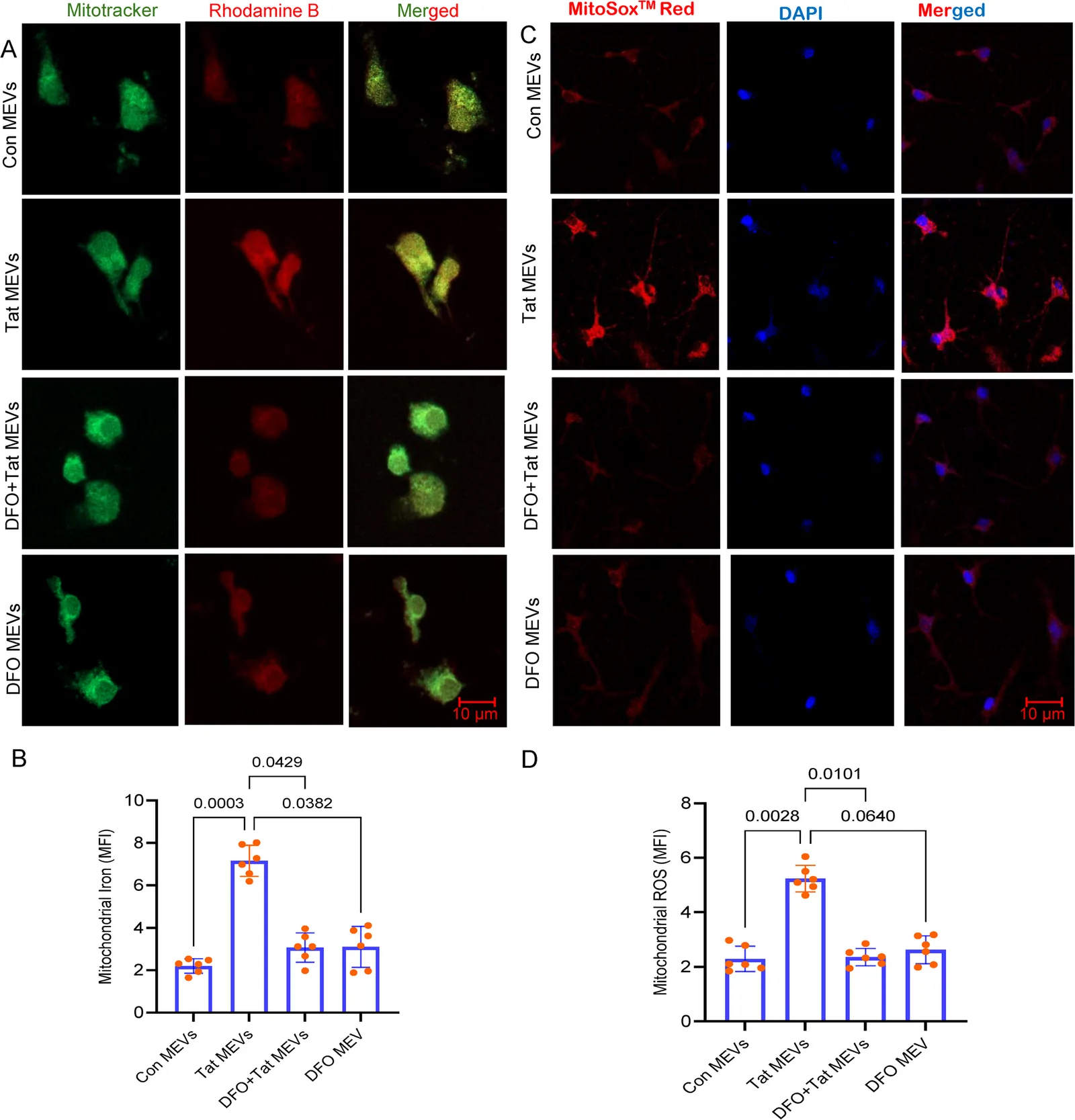
Effect of MEVs on mitochondrial iron and ROS: Representative images (**A**), and (**B**) quantification of mitochondrial iron levels in RHNs treated with Con MEVs, Tat MEVs, DFO + Tat MEVs, and DFO MEVs followed by staining with rhodamine B. Representative images (**C**), and (**D**) quantification of mitochondrial ROS levels in RHNs treated with Con MEVs, Tat MEVs, DFO + Tat MEVs, and DFO MEVs followed by staining with MitoSox red dye. Data are presented as mean ± SEM from 3 independent neuronal cultures, each treated with MEVs derived from 3 biological replicates of BV2 cells. A total of 6 ROI fields were analyzed across all replicates (2–3 neurons per field of biological replicates). Statistical analysis was performed using one-way ANOVA followed by Kruskal-Wallis post hoc test, with *P* < 0.05 considered statistically significant. DFO, deferoxamine; MEV, microglia-derived EVs; RHN, rat hippocampal neurons; ROI, region of interest; ROS, reactive oxygen species

Additionally, we assessed whether MEVs are localized to mitochondria in primary RHNs. MEVs were labeled with the EV-specific membrane dye ExoSparkler and applied to MitoTracker-stained neurons. Confocal imaging revealed clear colocalization of ExoSparkler-labeled MEVs (red fluorescence) with mitochondrial structures (green fluorescence), predominantly along the outer mitochondrial membrane and in regions proximal to the nucleus (Fig. 4C). Quantitative analysis using the Manders coefficient showed values ranging from 0.70 to 0.85 (Fig. 4D), indicating substantial overlap and suggesting that a significant proportion of MEVs reach and associate with neuronal mitochondria. Given that mitochondria can take up increased cytosolic Fe^2+^, we next evaluated mitochondrial Fe^2+^ levels using the rhodamine B dye and its subsequent effect on mitochondrial ROS using the MitoSox dye in RHNs exposed to either Con MEVs, Tat MEVs, DFO + Tat MEVs, and DFO MEVs for 48 h. As shown in Fig. 5A-D, exposure of RHNs to Tat-MEVs significantly increased both mitochondrial iron (Fig. 5A and B) and ROS (Fig. 5C and D) levels. Exposure of neurons to DFO + Tat MEVs or DFO MEVs alone normalized mitochondrial iron and ROS levels to those observed with Con MEVs.

**Fig. 6:**
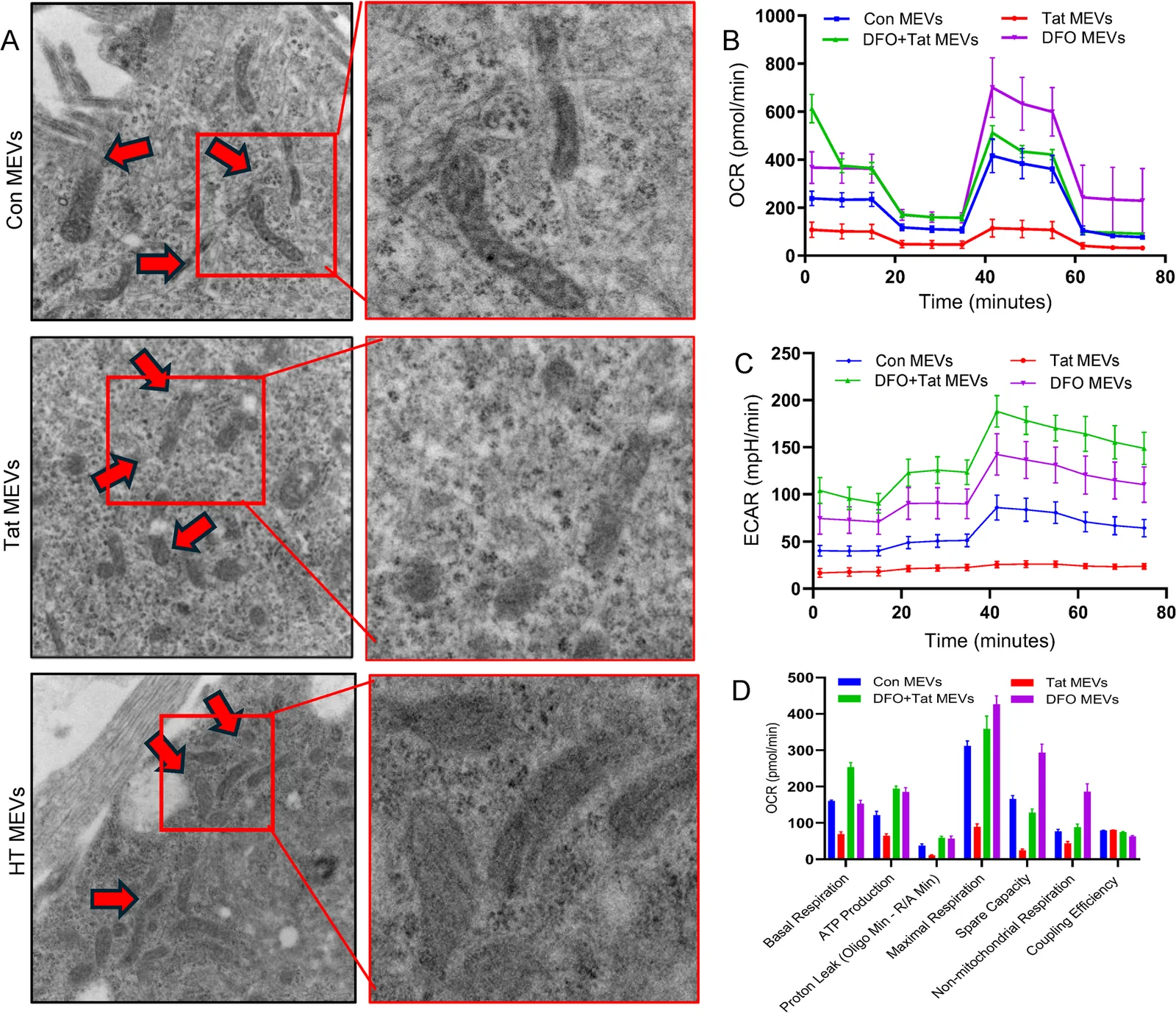
Effect of MEVs on mitochondrial structure and function: (**A**) Representative TEM images depicting mitochondrial structural changes in RHNs treated with Con MEVs, Tat MEV, and HT MEVs. Representative graph depicting quantifications of (**B**) OCR, (**C**) ECAR, and (**D**) respiration parameters in RHNs treated with Con MEVs, Tat MEV, DFO + Tat MEVs and DFO MEVs as estimated by Seahorse assay. Data are presented as mean ± SEM of 6 biological replicates. One-way ANOVA followed by Kruskal-Wallis post hoc test was performed. DFO, deferoxamine; ECAR, extracellular acidification rate; HT, heat-inactivated Tat; MEV, microglia-derived EVs; OCR, oxygen consumption rate; RHN, rat hippocampal neurons

Next, we sought to examine whether Tat MEVs could also impact mitochondrial structure and function. For this, we performed TEM analysis on RHNs exposed to Con MEVs, Tat MEVs, or HT MEVs for 48 h. RHNs exposed to Tat stimulated MEVs exhibited poorly defined mitochondrial membranes and cristae, unlike the neurons exposed to Con MEVs that had well preserved membranes and cristae (Fig. 6A). Assessment of mitochondrial function using the Seahorse assay further demonstrated that exposure of RHNs to Tat stimulated MEVs resulted in a significant decrease in the oxygen consumption rate (OCR) (Fig. 6B) as well as the extracellular acidification rate (ECAR) (Fig. 6C), which was normalized in neurons exposed to either DFO + Tat MEVs or DFO MEVs. Additionally, exposure of neurons to Tat MEVs was also shown to reduce basal respiration, ATP production, and spare respiratory capacity, although the coupling efficiency remained unchanged among the treatment groups (Fig. 6D).

### Tat MEVs altered the expression of synaptic proteins in rat hippocampal neurons

**Fig. 7:**
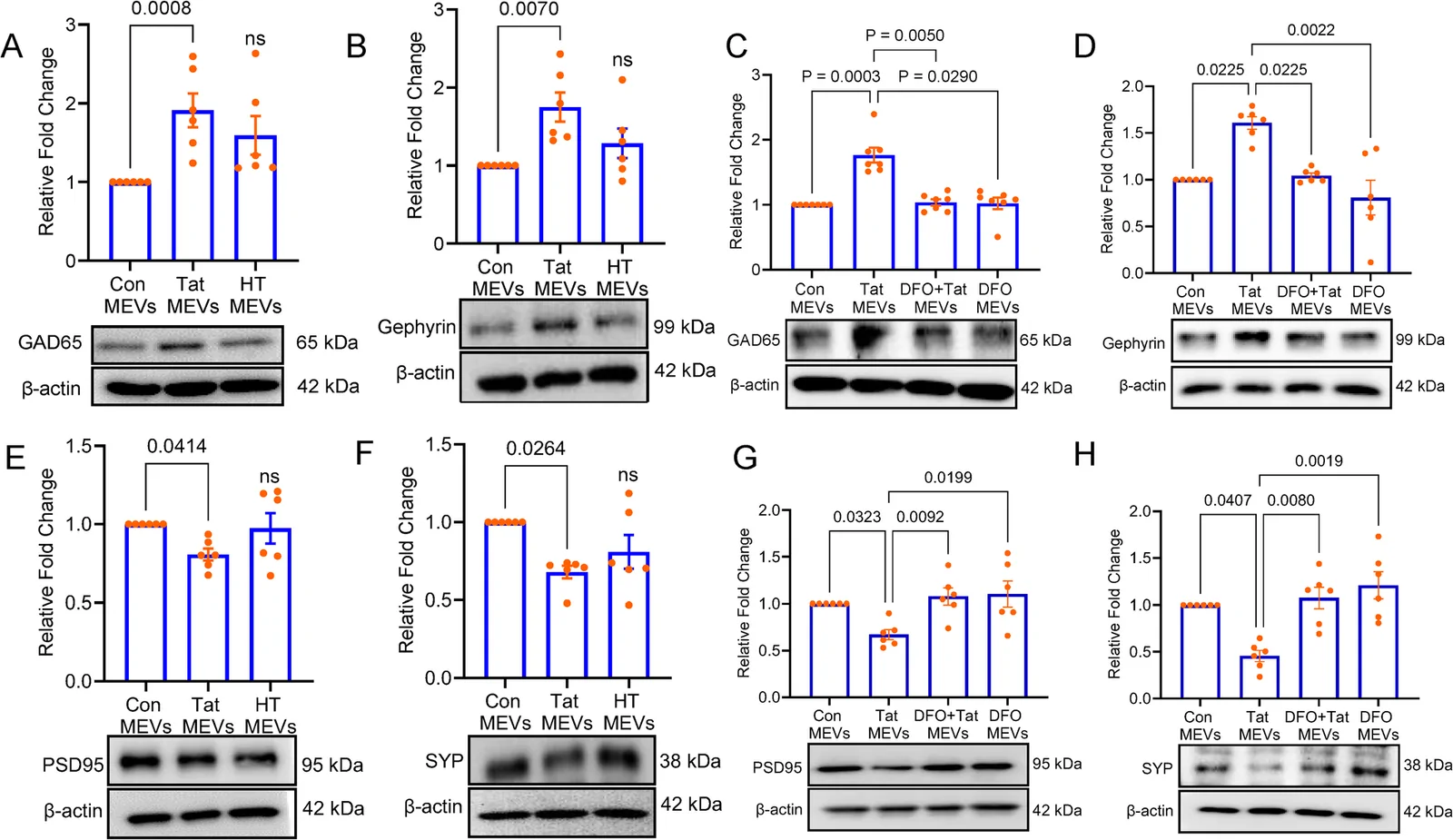
Effect of MEVs on neuronal synaptic proteins: Representative western blot images, and quantification of excitatory synaptic proteins (**A**-**D**) GAD65 and Gephyrin, and inhibitory synaptic proteins (**E**-**H**) PSD95 and synaptophysin in RHNs treated with Con MEVs, Tat MEV, HT MEVs, DFO + Tat MEVs and DFO MEVs. Data are presented as mean ± SEM of 6 biological replicates. One-way ANOVA followed by Kruskal-Wallis post hoc test was performed. *P* < 0.05 is considered statistical significance between treatment groups. DFO, deferoxamine; HT, heat-inactivated Tat; MEV, microglia-derived EVs; RHN, rat hippocampal neurons

Next, we evaluated the effects of Con MEVs, Tat MEVs, DFO + Tat MEVs, and DFO MEVs on the expression of synaptic proteins and dendritic structure in RHNs after 48 h of exposure. As shown in Fig. 7A and H, exposure of RHNs to Tat MEVs resulted in significantly increased expression of inhibitory postsynaptic proteins (GAD65 and gephyrin), while exposure to either DFO + Tat MEVs or DFO MEVs normalized the levels of these proteins to those of control cells.

Conversely, exposure of RHNs to Tat MEVs resulted in a significant downregulation of excitatory postsynaptic proteins (PSD95 and synaptophysin) compared to the control group.

Exposure of the cells to DFO + Tat or DFO MEVs, however, exhibited normalized protein levels.

### Tat MEVs induced synaptodendritic injury in rat hippocampal neurons

**Fig. 8:**
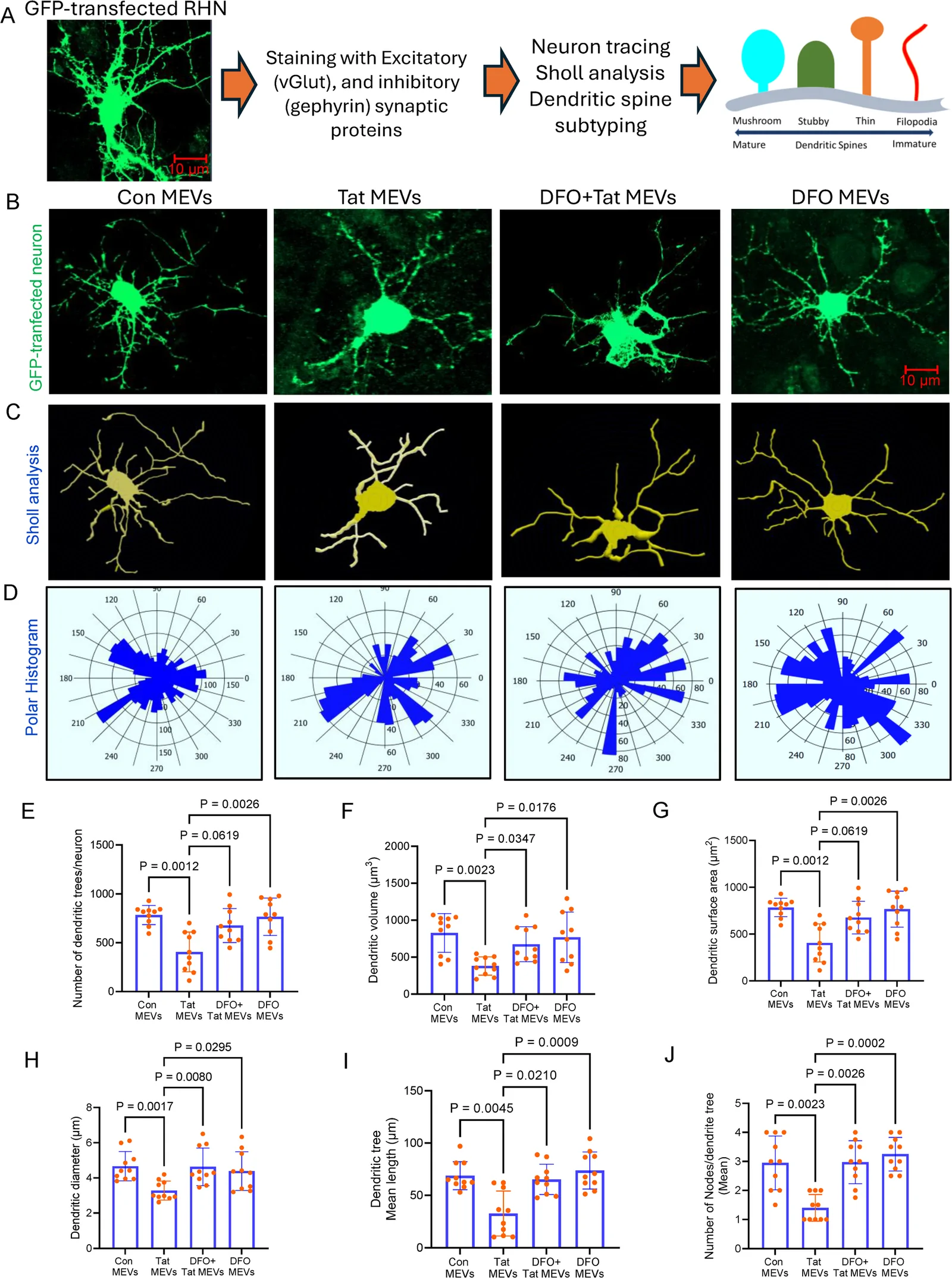
Effect of MEVs on neuronal morphology: (**A**) GFP-transfected neuron and workflow for the morphological analysis of neurons exposed to MEVs. **B** Representative confocal images showing dendritic arborization post exposure to MEVs. **C** Sholl analysis of neurons. **D** Polar histograms of neuronal dendrites. Quantitative analysis of (**E**) number of dendritic trees/neuron, (**F**) dendritic volume, (**G**) dendritic surface area, (**H**) dendritic diameter, (**I**) mean length of dendritic tree, and (**J**) mean number of nodes/dendritic tree. Data are presented as mean ± SEM. MEVs were derived from six biological replicates of BV2 cells and applied to primary neurons from six independent cultures derived from pups of six pregnant rats. A total of 10 neurons were traced and analyzed across all replicates, 1–2 neurons per biological replicate). One-way ANOVA followed by Kruskal-Wallis post hoc test was performed. *P* < 0.05 is considered statistical significance between treatment groups. DFO, deferoxamine; MEV, microglia-derived EVs; RHN, rat hippocampal neurons

Delving further into the synaptodendritic injury assessments, rat hippocampal neurons were transfected with a GFP-expressing plasmid and subsequently exposed to Con MEVs, Tat MEVs, DFO + Tat MEVs, or DFO MEVs for 48 h. Following treatment, the neurons were immunostained for excitatory (vGlut1) and inhibitory (gephyrin) synaptic markers (Fig. 8A).

**Fig. 9:**
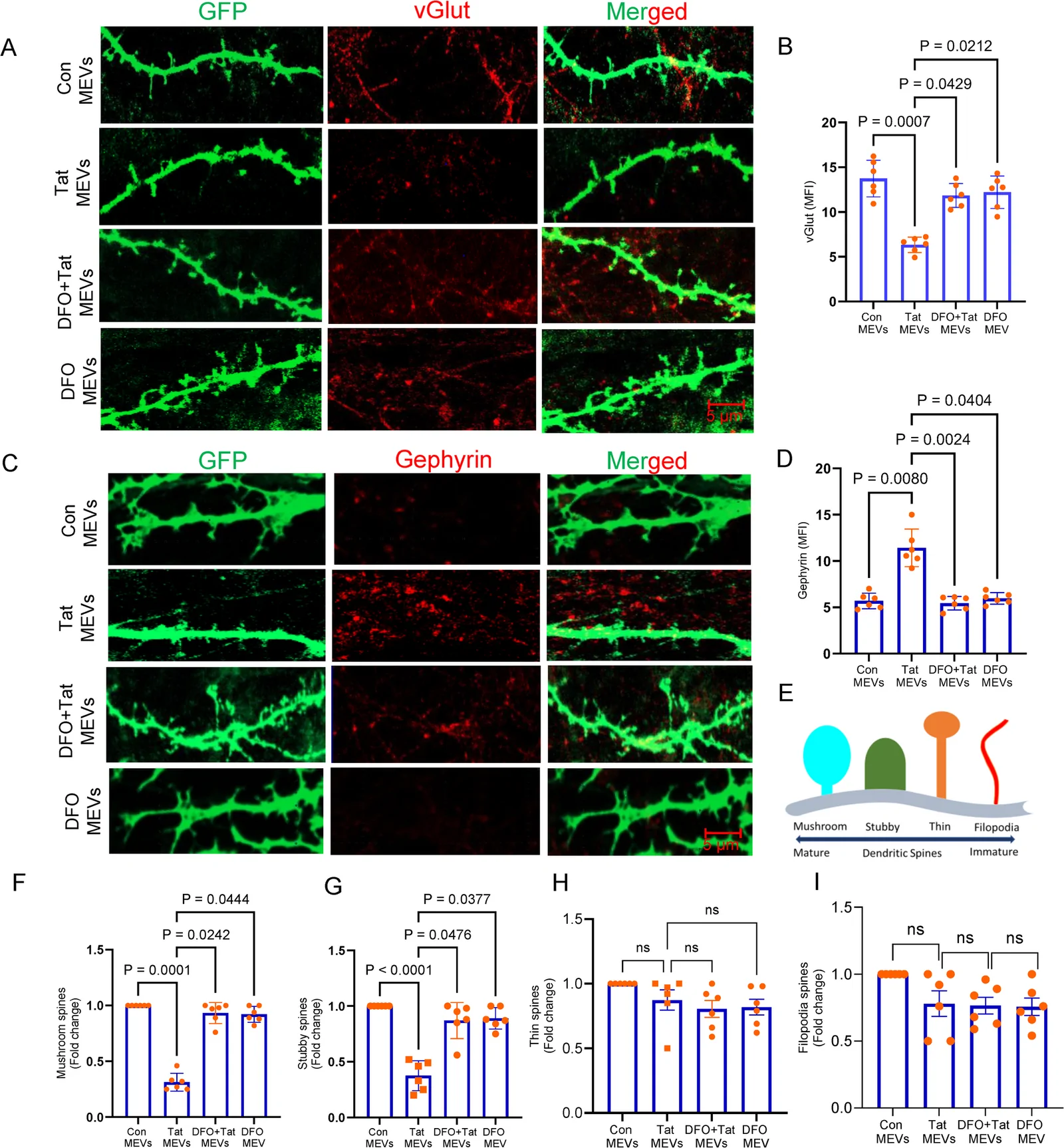
Effect of MEVs in synaptodendritic injury: Representative images depicting (**A**, **C**) neuronal dendritic spine structure, and staining with vGLut/gephyrin in RHNs exposed to Con MEVs, Tat MEV, DFO + Tat MEVs and DFO MEVs. Quantification of (**B**) vGLut, and (**D**) gephyrin in RHNs exposed to Con MEVs, Tat MEV, DFO + Tat MEVs and DFO MEVs. (**E)** Schematic showing different types of dendritic spines. Quantification of subtypes of spine (**F**) mushroom, (**G**) stubby, (**H**) thin, and (**I**) filopodia. Data are presented as mean ± SEM. MEVs were derived from six biological replicates of BV2 cells and applied to primary neurons from six independent cultures derived from pups of six pregnant rats. A total of 10 neurons were traced and analyzed across all replicates, 1–2 neurons per biological replicate). One-way ANOVA followed by Kruskal-Wallis post hoc test was performed. *P* < 0.05 is considered statistical significance between treatment groups. Sholl analysis for dendritic spine subtyping in RHNs exposed to Con MEVs, Tat MEV, DFO + Tat MEVs and DFO MEVs. DFO, deferoxamine; MEV, microglia-derived EVs; RHN, rat hippocampal neurons

**Fig. 10:**
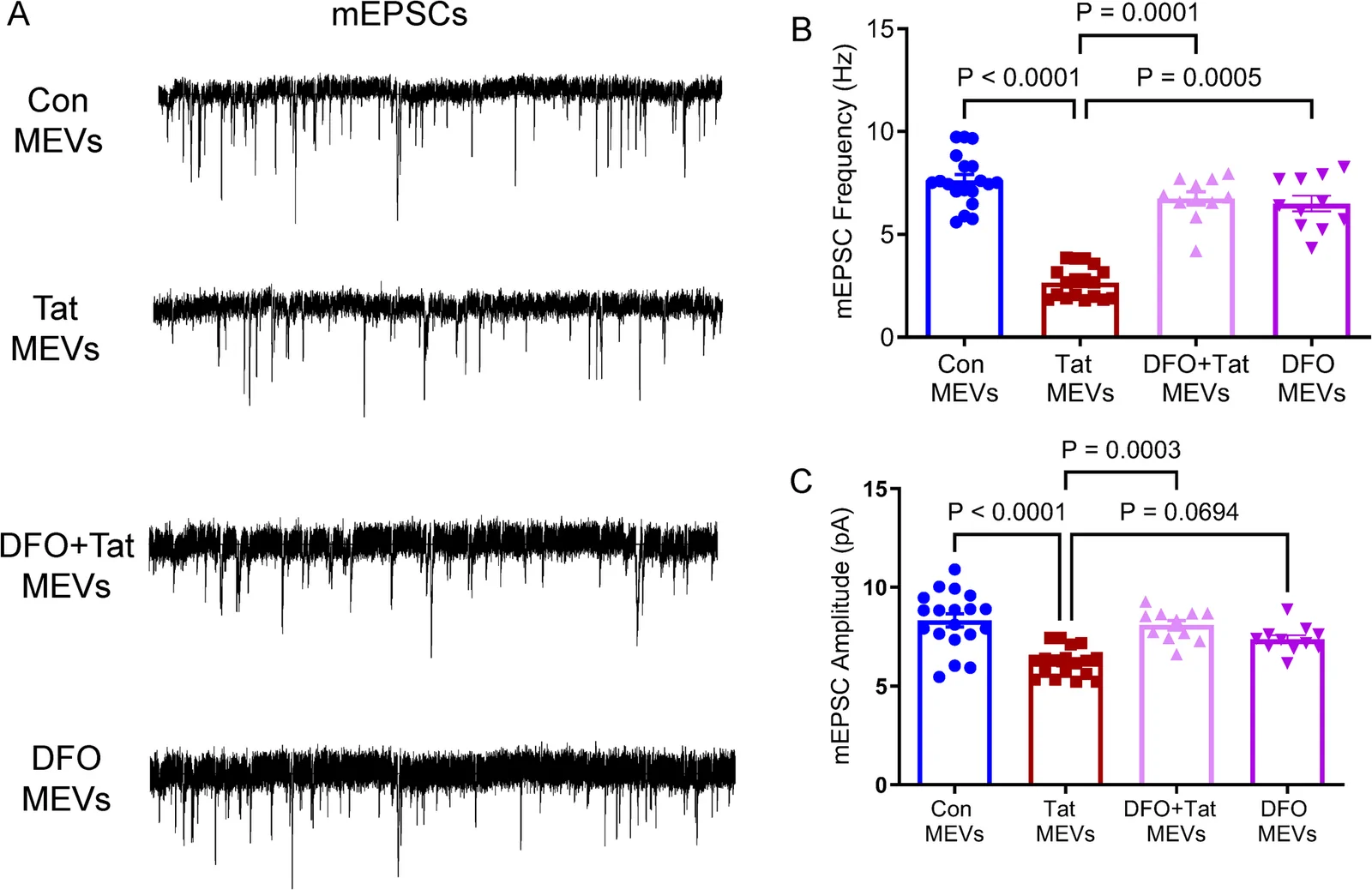
Effects of MEVs on neuronal function: (**A**) Representative electrophysiology traces of mEPSCs in neurons treated with Con MEVs, Tat MEVs, DFO + Tat MEVs, or DFO MEVs, with quantification of mEPSC (**B**) amplitude and (**C**) frequency. Data are presented as mean ± SEM from 6 independent neuronal cultures, each treated with EVs derived from six biological replicates of BV2 cells. A total of 10–15 neurons were traced and analyzed across all replicates (2–3 neurons per biological replicate). Statistical analysis was performed using one-way ANOVA followed by Kruskal-Wallis post hoc test, with *P* < 0.05 considered statistically significant. Abbreviations: DFO, deferoxamine; MEV, microglia-derived EVs; mEPSC, miniature excitatory postsynaptic currents; RHNs, rat hippocampal neurons

Next, GFP-transfected neurons were traced and subjected to Sholl analysis to assess alterations in dendritic arborization (Fig. 8B). The accompanying polar histogram illustrates the directional distribution of total dendritic length (Fig. 8C and D). Quantitative analysis revealed a significant reduction in multiple dendritic parameters, including the number of dendritic trees per neuron (Fig. 8E), dendritic volume (Fig. 8F), dendritic surface area (Fig. 8G), dendritic diameter (Fig. 8H), mean dendritic tree length (Fig. 8I), and the mean number of branch nodes per dendritic tree (Fig. 8J). However, neurons exposed to DFO + Tat MEVs or DFO MEVs showed a significant normalization of these parameters, indicating that iron chelation mitigates Tat MEV-induced dendritic impairment. As anticipated, dendritic spine density was preserved in neurons treated with Con MEVs but was markedly diminished following Tat MEV exposure. Notably, co-treatment with DFO + Tat MEVs or treatment with DFO MEVs alone improved dendritic spine density to levels comparable to those of the control group (Figs. 8E-J). We next sought to assess the number of mature spine subtypes (mushroom and stubby) in neurons exposed to various MEV groups. In RHNs exposed to Tat-MEVs, the number of mature spine subtypes (mushroom and stubby) was significantly decreased; however, no noticeable changes were observed in either the thin or filopodia spines in the presence of Tat-MEVs. In RHNs exposed to either DFO + Tat MEVs or DFO MEVs, the number of mature spine subtypes was comparable to Con MEV exposed RHNs (Fig. 9E-I). Furthermore, in RHNs exposed to Tat MEVs, there was significantly reduced expression of the excitatory synaptic protein vGlut1 (Fig. 9A-B) and increased expression of the inhibitory synaptic protein gephyrin (Fig. 9C-D), - an effect that was normalized in neurons exposed to either DFO + Tat MEVs or DFO MEVs (Fig. 9A-Figure 9D). Electrophysiology recordings were also performed to assess neuronal functioning following exposure to either Con MEVs, Tat MEVs, DFO + Tat MEVs, or DFO MEVs. As shown in Fig. 10A-C, RHNs exposed to Tat- stimulated MEVs exhibited reduced frequency and amplitude of miniature excitatory postsynaptic currents (mEPSCs), and this was normalized in RHNs exposed to either DFO + Tat MEVs or DFO MEVs.

## Discussion

The use of highly effective cART has significantly extended the lifespan of PLWH, transforming AIDS into a chronic, manageable condition [50]. Despite this progress, many PLWH continue to experience cognitive, motor, and behavioral impairments collectively known as HAND [51, 52]. The pathogenesis of HAND has also been linked to oxidative stress, organelle dysfunction, synaptodendritic damage, and neuroinflammation [7, 19, 53–58]. Viral proteins such as HIV Tat and gp120 are critical contributors to these processes, primarily through their roles in iron dysregulation and synaptic injury [7, 21, 59–61], ultimately culminating in neuroinflammation.

Both HIV Tat and gp120 have been shown to disrupt endolysosomal function, leading to the release of iron into the cytosol and thereby compromising the integrity of the endolysosomal membrane [17, 20–23]. This disruption can enhance mitochondrial ROS production, leading to cell death via apoptosis and/or ferroptosis, thus suggesting a link between ferroptosis-induced neurodegeneration and NeuroHIV [24, 25].

EVs, including exosomes and microvesicles, have emerged as critical mediators of intercellular communication in the CNS and play a significant role in the neuropathogenesis of NeuroHIV [41, 62–65]. Studies have also shown that microglia-derived large EVs carrying β‑amyloid, when injected into the mouse entorhinal cortex, propagate synaptic dysfunction, disrupt cortical-hippocampal network activity, and impair memory, mirroring features of Alzheimer’s disease [66].

EVs derived from HIV-infected or HIV Tat-exposed glial cells, particularly microglia and astrocytes, have been shown to carry viral proteins, inflammatory mediators, and neurotoxic cargo, which, in turn, contribute to synaptic dysfunction, neuronal injury, and neuroinflammation [38–41]. Our previous studies have shown that EVs released from astrocytes and microglia in response to HIV Tat contribute to synaptic dysfunction and neuroinflammation [39, 40]. In another study, we demonstrated that HIV Tat induces ferroptosis in microglia by modulating key regulators, such as ACSL4 and FTH1, along with a reduction in GPX4 expression [43]. The ferroptosis-associated molecules could also be packaged into Tat-induced MEVs, which in turn can propagate neuronal injury through iron-dependent ferroptotic mechanisms.

In this study, we observed that exposure of BV2 microglial cells to HIV-1 Tat resulted in increased expression of several key ferroptosis-associated proteins, including TF, TFR1, STEAP3, DMT1, and FTH1. Importantly, pretreatment with the iron chelator DFO markedly attenuated these Tat-induced elevations, indicating that Tat-mediated dysregulation of iron-handling pathways is at least partially dependent on intracellular iron availability. In parallel, analysis of EV biogenesis-related transcripts, including smpd3, tsg101, pdcd6ip, and rab27a, revealed no significant differences in DFO-pretreated BV2 cells compared with control cells.

These findings suggest that DFO does not directly influence EV biogenesis gene expression under our experimental conditions, supporting the interpretation that the protective effects of DFO are primarily mediated through suppression of Tat-induced iron-dependent ferroptotic signaling rather than through modulation of EV production machinery.

In addition, we observed robust internalization of ExoSparkler-labeled MEVs by rat primary neurons, confirming efficient vesicle uptake. We further found that HIV Tat-activated MEVs from BV2 cells significantly upregulated the expression of TF, TFR1, STEAP3, DMT1, and FTH1 in RHNs and RCNs compared to neurons exposed to either Con MEVs or HT MEVs.

Consistent with our previous findings, these MEVs ranged from 50 to 200 nm [39] and were shown to carry TF, TFR1, and FTH1 cargoes. The role of iron in this process was further validated in RHNs and RCNs exposed to EVs derived from BV2 microglial cells pre-treated with the iron chelator DFO. Exposure of neurons to these MEVs resulted in a marked reduction in the expression of key iron-regulatory proteins TF, TFR1, STEAP3, DMT1, and FTH1. A potential concern in EV-based studies is that soluble components present in the conditioned media could co-isolate with EV preparations and affect downstream biological outcomes. To address this concern, we conducted additional control experiments in parallel, wherein neurons were also exposed to the corresponding non-EV fractions collected during the EV isolation process for each experimental condition. These non-EV fractions, representing the soluble components remaining after EV removal were processed using the same workflow as the EV samples.

Notably, the neuronal responses observed following exposure to Tat-MEVs or DFO-MEVs were not recapitulated by the corresponding non-EV fractions, indicating that these effects were predominantly mediated by EV-associated cargo rather than residual soluble components. While a medium replacement step prior to EV collection could further minimize potential contamination by residual treatment compounds, the inclusion of these non-EV controls provides an important safeguard and strengthens the interpretation of EV-mediated signaling in our study.

These findings demonstrate that Tat-MEV uptake is not merely a passive event, but instead results in functionally relevant neuronal injury. The significant elevation in LDH release following Tat-MEV exposure indicated compromised membrane integrity and cytotoxic damage, supporting the idea that Tat-MEVs carried the neurotoxic cargo that was capable of inducing neuronal death. Importantly, the reduced cytotoxicity in neurons treated with DFO + Tat MEVs suggested that iron-dependent mechanisms contributed substantially to Tat-MEV-mediated neurotoxicity. This protective effect aligns with our observed improvements in synaptic and mitochondrial functional outcomes and supports the involvement of iron-dependent oxidative injury pathways. While the observed changes are consistent with cellular stress responses frequently associated with ferroptosis, LDH release alone does not distinguish ferroptotic cell death from other forms of neuronal injury. Therefore, our findings indicate towards the occurrence of iron-associated neurotoxicity and ferroptosis-associated stress. These findings further demonstrate that the effects of Tat-MEVs extend beyond sublethal synaptic and mitochondrial dysfunction, resulting in measurable neuronal injury.

Additionally, exposure of RHNs to Tat-MEVs was also shown to increase endolysosomal pH and lysosomal membrane permeability, consistent with previous reports of endolysosomal deacidification by HIV Tat [21]. Deacidification was found to be accompanied by decreased endolysosomal iron levels and increased cytosolic iron, in turn, leading to enhanced ROS production. These findings align with previous reports, which have shown that endolysosomal deacidification leads to the release of divalent metal cations, such as iron and calcium, into the cytosol [22, 23]. The elevated cytosolic iron likely contributed to increased mitochondrial iron levels and ROS production through Fenton’s reaction, which were associated with mitochondrial structural and functional impairments. Mitochondrial changes were found to be mitigated in neurons exposed to DFO + Tat MEVs, and DFO MEVs thereby supporting a critical role for iron dysregulation in mediating Tat-MEV-induced neuronal injury. Together, these findings suggest that EV-mediated disruption of intracellular iron homeostasis contributes to oxidative stress and organellar dysfunction, processes that are commonly associated with ferroptosis-related cellular stress. Additionally, we observed colocalization of ExoSparkler-labeled MEVs with mitochondria (MitoTracker-stained), both at the membrane and in regions proximal to the nucleus, which supports the possibility of targeted delivery of iron-laden cargo into mitochondrial compartments.

Furthermore, exposure of RHNs to Tat-MEVs was shown to significantly alter the expression of synaptic proteins, increasing the expression of inhibitory synaptic proteins (GAD65 and gephyrin) and decreasing the expression of excitatory synaptic proteins (PSD95 and synaptophysin). Exposure of RHNs to DFO MEVs or DFO + Tat MEVs normalized the levels of these proteins. Additionally, RHNs exposed to Tat MEVs also showed decreased mEPSCs, thus suggesting functional impairment of the neurons. Concomitantly, Tat MEV exposure resulted in reduction in dendritic arborization of the neurons, which was normalized with exposure of neurons with DFO MEVs or DFO + Tat MEVs. Additionally, Tat MEV exposure of neurons also resulted in a reduction of dendritic spine density, particularly affecting the mature spine subtypes (mushroom and stubby), with no significant effect on thin or filopodia spines. Previous studies have demonstrated that HIV infection is associated with damage to neocortical pyramidal neurons and disruptions in excitatory neurotransmission, including reduced expression of the postsynaptic density protein PSD95 [7, 67]. Additionally, exposure to the HIV Tat protein has been linked to increased expression of the inhibitory synaptic protein gephyrin, indicating a shift toward enhanced inhibitory signaling in the brain [68]. Studies in HIV transgenic mice have also shown increased expression of gephyrin (associated with inhibitory synaptic transmission) and reduced number of apical dendritic spines, thus highlighting the role of HIV Tat in synaptic disruption and altered dendritic architecture [8]. Likely, alterations in the balance of excitatory/inhibitory proteins could thus underline the neuronal structural and functional impairments observed in NeuroHIV. Exposure of RHNs to DFO + Tat MEVs, and DFO MEVs, normalized the excitatory and inhibitory synaptic protein levels, as well as the numbers of mature spine subtypes, thus suggesting a protective effect against Tat MEV-induced synaptodendritic injury and underscoring the potential role of iron chelation as a possible therapeutic strategy.

Several limitations of this study should be acknowledged. First, although our findings demonstrate EV-mediated transfer of iron-handling cargo and establish a link between neuronal iron dysregulation, oxidative stress, mitochondrial dysfunction, and synaptodendritic injury, they do not conclusively establish ferroptotic cell death in recipient neurons. Canonical ferroptosis-specific endpoints, including lipid peroxidation measurements, GPX4 depletion, ACSL4 dependence, and pharmacological rescue with ferroptosis inhibitors such as ferrostatin-1 or liproxstatin-1, were not evaluated in the current study and warrant investigation in future work. Accordingly, the observed neuronal phenotypes are most appropriately interpreted as ferroptosis-associated stress responses and iron-dependent neurotoxicity. Second, although DFO pretreatment attenuated the neurotoxic effects of Tat-MEVs, DFO likely influences multiple cellular pathways and EV cargo populations in donor microglia. Therefore, the present experiments do not establish a direct causal role for individual cargo molecules such as TF, TFR1, or FTH1. Future studies employing cargo-specific genetic manipulation approaches will be necessary to define the relative contribution of individual EV-associated iron-regulatory proteins. Finally, while the BV2 microglial cell line provides a practical and widely used model for mechanistic investigations, it does not fully recapitulate the complexity of primary microglia or human microglial biology. Validation of these findings in primary microglial cultures and in vivo NeuroHIV models will be important for establishing their translational relevance.

**Fig. 11:**
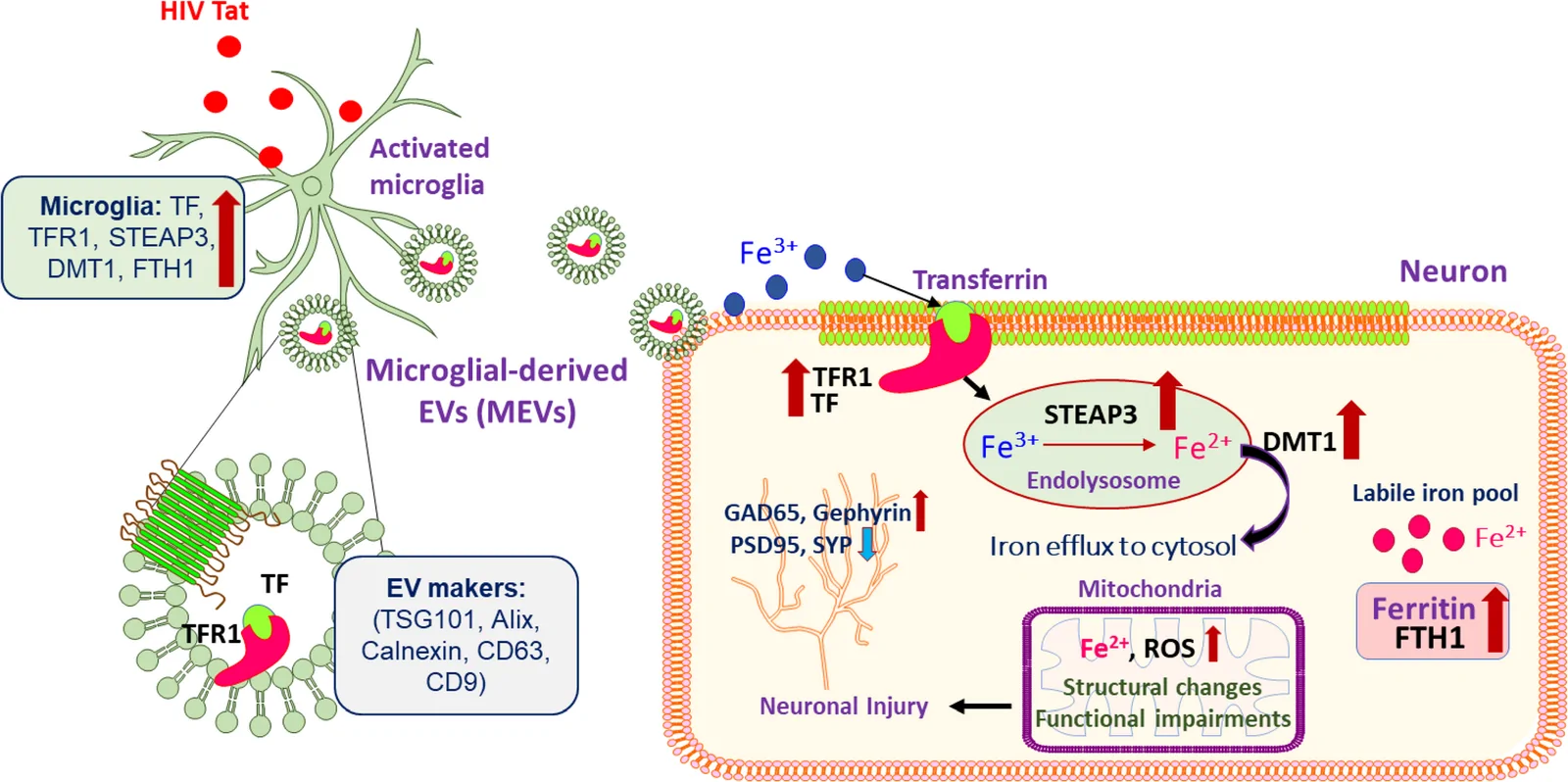
Schematic summarizing that HIV Tat-activated microglia-derived extracellular vesicles (Tat-MEVs) mediate neuronal injury via the ferroptosis-associated stress pathway. This study demonstrates that HIV Tat induces the release of MEVs enriched with TF, TFR1, and FTH1. Neuronal uptake of these MEVs disrupts intracellular iron homeostasis by promoting iron efflux from endolysosomes into the cytosol, leading to elevated cytosolic iron and increased ROS. This dysregulation drives mitochondrial iron overload and ROS production, resulting in mitochondrial dysfunction and synaptodendritic injury, as evidenced by reduced dendritic spine density, and reduced mEPSCs. DMT1, divalent metal transporter 1; FTH1, ferritin heavy chain 1; mEPSCs, miniature excitatory postsynaptic currents; MEV, microglia-derived EVs; ROS, reactive oxygen species; STEAP3, six-transmembrane epithelial antigen of prostate 3; TF, transferrin; TFR1, transferrin receptor 1

In summary (Fig. 11), HIV Tat induces the release of MEVs enriched in TF, TFR1, and FTH1.

Upon neuronal uptake, these MEVs disrupt iron homeostasis, leading to oxidative stress, impairment of endolysosomal and mitochondrial function. These disturbances culminate into synaptodendritic injury and neuronal dysfunction, as evidenced by alterations in synaptic protein expression, reduced dendritic spine integrity, and changes in mEPSCs. The protective effects observed following iron chelation support a central role for iron-dependent mechanisms in mediating these outcomes. Collectively, these findings identify a novel microglia-to-neuron EV signaling pathway driving neuronal iron dysregulation and neurotoxicity in the context of HIV Tat exposure. While the observed molecular and cellular changes are consistent with ferroptosis-associated stress pathways, definitive confirmation will require assessment using canonical ferroptotic specific endpoints in recipient neurons. Overall, this work establishes a framework for EV-mediated iron dysregulation in HIV-associated neurocognitive disorders and highlights iron-targeted strategies as a potential therapeutic avenue.

## Supplementary Information

Supplementary Material 1: Supplementary Fig 1. Effect of HIV Tat on iron-regulatory proteins in BV2 microglial cells: Representative western blots and quantification showing the expression of iron-regulatory proteins (A) TF, (B) TFR1, (C) STEAP3, (D) DMT1 and (E) FTH1 in control BV2 cells and BV2 cells exposed to HIV Tat or HT; (F) TF, (G) TFR1, (H) STEAP3, (I) DMT1 and (J) FTH1 in BV2 cells pretreated with DFO. Graphs showing mRNA levels of EV biogenesis markers (K) smpd3, (L) TSG101, (M) pdcd6ip, and (N) rab27a. Data are presented as mean ± SEM of 6 biological replicates. One‐way ANOVA followed by Kruskal-Wallis post hoc test was performed. P<0.05 is considered statistical significance between treatment groups. DMT1, divalent metal transporter 1; FTH1, ferritin heavy chain 1; MEV, microglia-derived EVs; RCN, rat cortical neurons; RHN, rat hippocampal neurons; STEAP3, six-transmembrane epithelial antigen of prostate 3; TF, transferrin; TFR1, transferrin receptor 1.

Supplementary Material 2: Supplementary Figure 2. Characterization of MEVs: (A) Workflow for the isolation of EVs from conditioned media of BV2 cells exposed to HIV Tat. (B) Quantification of EV numbers by ZetaView. (C) Size distribution of MEVs. (D) Representative western blots showing expression of EV markers in BV2 MEVs (E) Representative western blots showing expression of iron-regulatory proteins (TF, TFR1, & FTH1) in MEV cargoes. (F) Quantification of EV numbers for BV2 cells pretreated with DFO. (G) Representative western blots showing expression of EV markers in DFO MEVs. (H) Representative western blots showing expression of iron-regulatory proteins (TF, TFR1, & FTH1) in DFO MEV cargoes. Data are presented as mean ± SEM of 6 biological replicates. One‐way ANOVA followed by Sidak’s post hoc test was performed. P<0.05 is considered statistical significance between treatment groups. DMT1, divalent metal transporter 1; FTH1, ferritin heavy chain 1; RHN, rat hippocampal neurons; STEAP3, six-transmembrane epithelial antigen of prostate 3; TF, transferrin; TFR1, transferrin receptor 1.

Supplementary Material 3: Supplementary Figure 3. Effect of MEVs, and NEFs on neurons: Representative western blots and quantification showing the expression of iron-regulatory proteins (A) TF, (B) FTH1 in RHNs, and (C) TF, (D) FTH1 in RCNs treated with Con MEVs, Tat MEVs, DFO+Tat MEVs, or DFO MEVs, Con NEF, Tat NEF, DFO+Tat NEF, or DFO NEF. Data are presented as mean ± SEM of 3 biological replicates each with two technical replicates thar are treated with MEV, and NEF isolates form three biological replicates of BV2 cells. One‐way ANOVA followed by Kruskal-Wallis post hoc test was performed. P<0.05 is considered statistically significant between treatment groups. FTH1, ferritin heavy chain 1; RCN, rat cortical neurons; RHN, rat hippocampal neurons; TF, transferrin.

Supplementary Material 4: Supplementary Figure 4. Characterization of GW4869 MEVs: (A) Quantification of EV numbers by ZetaView. (B) Representative western blot showing expression of iron-regulatory proteins TF, TFR1, and FTH1 in MEV cargoes. Representative western blot images showing expression of iron-regulatory proteins TF, TFR1, STEAP3, DMT1, & FTH1 in (C-G) RCNs & (H-L) RHNs treated with Con MEVs, Tat MEVs, and GW+Tat MEVs, and GW MEVs. DFO, deferoxamine; DMT1, divalent metal transporter 1; FTH1, ferritin heavy chain 1; GW, GW4869; MEV, microglia-derived EVs; RCNs, rat cortical neurons; RHNs, rat hippocampal neurons; STEAP3, six-transmembrane epithelial antigen of prostate 3; TF, transferrin; TFR1, transferrin receptor 1. Data are presented as mean ± SEM of 6 biological replicates. One‐way ANOVA followed by Kruskal-Wallis post hoc test was performed. P<0.05 is considered statistical significance between treatment groups. DMT1, divalent metal transporter 1; FTH1, ferritin heavy chain 1; RHN, rat hippocampal neurons; STEAP3, six-transmembrane epithelial antigen of prostate 3; TF, transferrin; TFR1, transferrin receptor 1.

Supplementary Material 5.

## Data Availability

The datasets used and/or analyzed during the current study are available from the corresponding authors on reasonable request.
